# Chromatin landscape, DSB levels, and cKU-70/80 contribute to patterning of meiotic DSB processing along chromosomes in *C*. *elegans*

**DOI:** 10.1371/journal.pgen.1010627

**Published:** 2023-01-27

**Authors:** Laura I. Lascarez-Lagunas, Marina Martinez-Garcia, Saravanapriah Nadarajan, Brianna N. Diaz-Pacheco, Elizaveta Berson, Mónica P. Colaiácovo

**Affiliations:** Department of Genetics, Blavatnik Institute, Harvard Medical School, Boston, Massachusetts, United States of America; University of Delaware, UNITED STATES

## Abstract

Programmed DNA double-strand break (DSB) formation is essential for achieving accurate chromosome segregation during meiosis. DSB repair timing and template choice are tightly regulated. However, little is known about how DSB distribution and the choice of repair pathway are regulated along the length of chromosomes, which has direct effects on the recombination landscape and chromosome remodeling at late prophase I. Here, we use the spatiotemporal resolution of meiosis in the *Caenorhabditis elegans* germline along with genetic approaches to study distribution of DSB processing and its regulation. High-resolution imaging of computationally straightened chromosomes immunostained for the RAD-51 recombinase marking DSB repair sites reveals that the pattern of RAD-51 foci throughout pachytene resembles crossover distribution in wild type. Specifically, RAD-51 foci occur primarily along the gene-poor distal thirds of the chromosomes in both early and late pachytene, and on both the X and the autosomes. However, this biased off-center distribution can be abrogated by the formation of excess DSBs. Reduced condensin function, but not an increase in total physical axial length, results in a homogeneous distribution of RAD-51 foci, whereas regulation of H3K9 methylation is required for the enrichment of RAD-51 at off-center positions. Finally, the DSB recognition heterodimer cKU-70/80, but not the non-homologous end-joining canonical ligase LIG-4, contributes to the enriched off-center distribution of RAD-51 foci. Taken together, our data supports a model by which regulation of the chromatin landscape, DSB levels, and DSB detection by cKU-70/80 collaborate to promote DSB processing by homologous recombination at off-center regions of the chromosomes in *C*. *elegans*.

## Author summary

Accurate chromosome segregation during meiosis is essential for all sexually reproducing organisms. Errors during meiosis can result in infertility, stillbirths, miscarriages, and birth defects. Therefore, understanding how different steps of meiosis are regulated is of vital importance to reproductive health. One such key step is the formation of programmed DNA double-strand breaks (DSBs), which are required for recombination between homologous chromosomes. DSBs are a source of genetic variability but also of genome instability; thus, regulating their formation and correct repair is paramount. In the present study, we identify different factors regulating the distribution of meiotic DSB repair sites marked by the RAD-51 recombinase along the chromosomes in the nematode *Caenorhabditis elegans*. We find that either excess levels of breaks or alterations in chromosome compaction can produce a homogeneous distribution of RAD-51 foci along the chromosome length. Moreover, we find that biased RAD-51 foci distribution to the terminal thirds of the chromosomes in wild type worms is regulated by the epigenetic landscape and DNA repair proteins. Our study reveals a variety of factors involved in the complex regulation of DSB distribution and repair by homologous recombination during meiosis in *C*. *elegans*.

## Introduction

Meiosis is the specialized cell division program that produces haploid gametes for sexual reproduction by halving the number of chromosomes. This is achieved by accurately segregating homologous chromosomes (homologs) to opposite poles of the cell at meiosis I and sister chromatids at meiosis II. A series of unique steps during prophase I ensure accurate chromosome segregation at meiosis I, including pairing and synapsis between homologs and programmed meiotic DNA double-strand break (DSB) formation followed by repair via homologous recombination (HR) [1]. These events occur in the context of fully replicated chromosomes comprised of pairs of sister chromatids organized with chromatin loops emanating from central chromosome axes. DSBs are generated at chromatin loops by the Spo11 protein [2], and tethering of these loops to the axes facilitates repair by interhomolog recombination [3]. These interhomolog recombination events can lead to the formation of crossovers (COs) resulting in physical attachments (chiasmata), underpinned by flanking sister chromatid cohesion, that hold the pairs of homologs (bivalents) together ensuring sufficient tension at the metaphase I plate and subsequent accurate chromosome segregation at meiosis I. An excess number of DSBs, relative to the number of COs, is observed along chromosomes during meiosis in most species, most likely to ensure that at least one CO event is produced between each pair of homologs by the HR pathway [4]. This phenomenon, referred to as “CO assurance”, is achieved by repressing DSB repair with the sister chromatid and with other non-homology directed pathways [5]. *C*. *elegans*, which has holocentric chromosomes, exhibits complete CO interference [6,7]. Therefore, a single CO is formed between each pair of homologous chromosomes. This single CO event takes place at an off-center position corresponding to the terminal thirds of the total length of the chromosomes (regions referred to as the arms) [8,9]. This off-center CO placement initiates the late prophase I chromosome remodeling process that results in the formation of asymmetric cruciform-shaped bivalents consisting of perpendicularly intersecting long and short arms that ensure the proper segregation of each homolog to opposite poles at meiosis I [10,11]. The establishment of the short and long arm domains is key for the relocalization of different proteins that participate in kinetochore formation and the maintenance of sister chromatid cohesion along the long arm of the bivalent. Several mechanisms ensure this differential localization of proteins [10], but how CO formation is biased to off-centered positions, and whether that decision is being made at the CO designation level or earlier during formation or processing of DSBs, is still unclear. Therefore, understanding how DSB levels and their distribution along the length of the chromosomes are regulated is of special importance.

Most organisms have recombination events located at the distal regions of the chromosomes [9,12–14]. The number of meiotic DSBs vary widely, whereas COs are usually limited to 1–3 events per bivalent [15]. Many factors have been associated with the restriction of DSB formation including inhibition by *cis* or *trans* interference partially regulated by the ATM/ATR pathway, competition for different repair factors, HORMA domain-containing meiotic axial proteins, and epigenetic regulators [16]. Moreover, most organisms undergo programmed meiotic DSBs at specific locations in the genome referred to as hotspots that share certain characteristics: they are nucleosome low density regions linked to active transcription, open chromatin locations, promoter or intergenic zones, or regions associated with DNA specific sequence motifs [16]. However, worms differ in some of these aspects. For instance, *C*. *elegans* lacks DSB hotspots, and recombination takes place in gene-depleted regions (the terminal thirds of the chromosomes) [17,18]. In addition, although chromatin-associated proteins seem to play a role in regulating DSB levels [19–21,10], it is unknown how or which histone marks can influence the number of DSBs being formed. Furthermore, DSB formation and recombination on the sex chromosomes is differentially regulated with respect to the autosomes throughout species [22], and the X chromosome in *C*. *elegans* also exhibits some of these distinct characteristics. In hermaphrodites (XX), the X chromosomes are more condensed than the autosomes, remain transcriptionally-repressed until late meiotic prophase I, undergo lower levels of DSBs, and exhibit a less pronounced repression in recombination levels at the center region compared to the autosomes [9,23,24]. Finally, unrepaired DSBs present at the late pachytene stage of prophase I can be repaired by HR-independent pathways [25,26]. Additionally, it is unclear whether DSB distribution is altered depending on whether they are formed early or late in pachytene.

Despite its importance for chromosome segregation and gamete viability, how meiotic DSB distribution and repair processing along chromosomes are regulated is poorly understood. In this study, we take advantage of the spatiotemporal arrangement of nuclei in the *C*. *elegans* gonad as well as the small number and size of its chromosomes to investigate the frequency and distribution of DSB repair sites marked by RAD-51 foci along autosomes and X chromosomes. We also determine how patterning of DSB processing is regulated at different stages of pachytene after either endogenous or exogenous DSB formation, and in mutants for genes required in chromatin regulation and DSB repair. We expand upon our prior finding that DSB repair sites, marked by RAD-51 foci, are preferentially localized on the arms of the chromosomes [27], and uncover different levels of regulation depending on the number of breaks formed, chromatin compaction, epigenetic regulation, and DNA repair proteins, that ensure that DSBs are processed to form recombination events at the distal thirds of the chromosomes in *C*. *elegans*.

## Results

### The X chromosome and the autosomes exhibit a biased off-center distribution of RAD-51 foci throughout pachytene that is lost upon excess DSB formation

**Fig 1:**
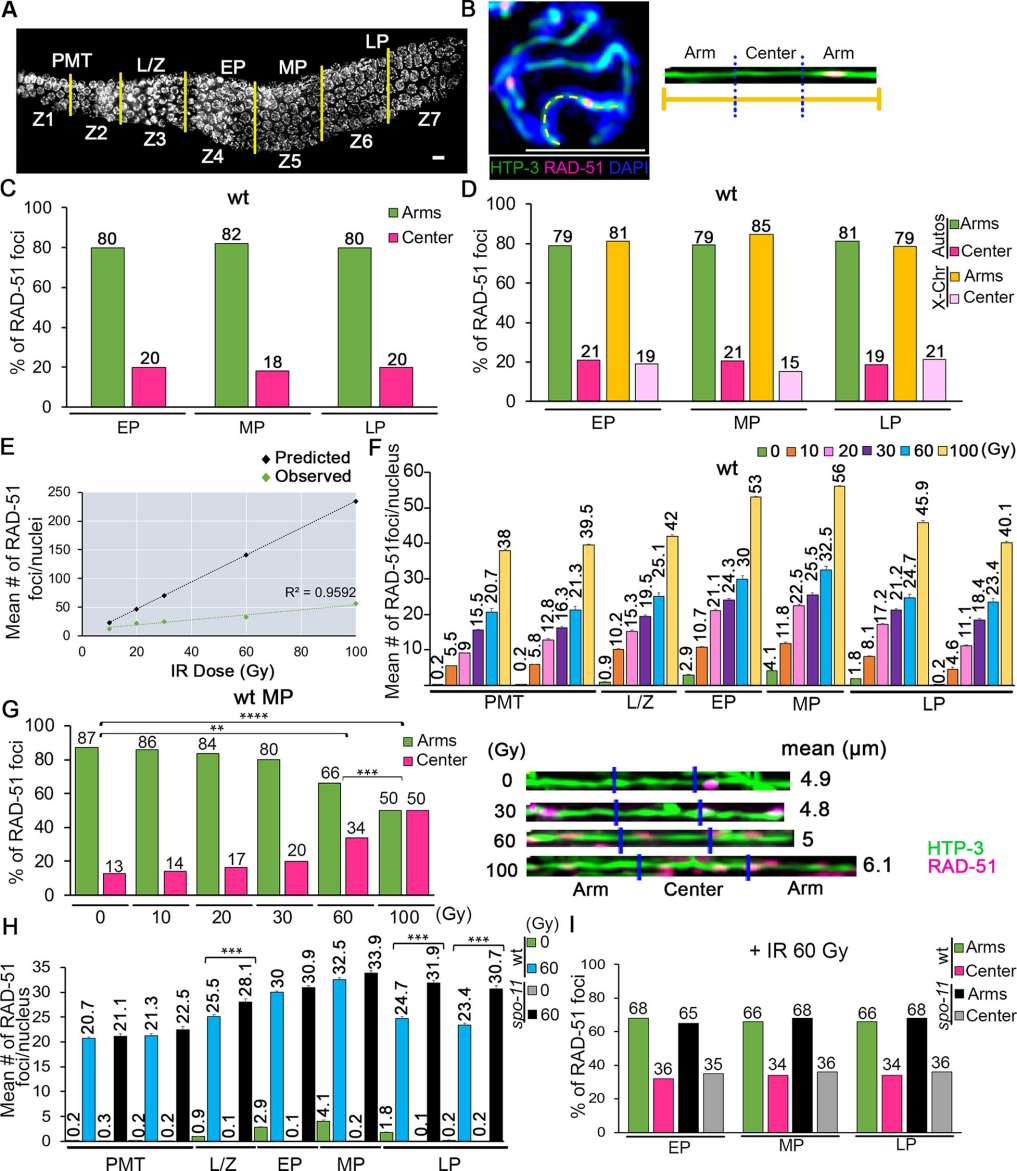
Distribution of meiotic RAD-51 foci is biased to the arms of the chromosomes throughout pachytene in normal conditions. **(A)** High-resolution image of a whole-mounted wild type hermaphrodite gonad stained with DAPI oriented from left to right. The different stages of meiotic prophase are indicated above the gonad and the seven equally sized zones scored for RAD-51 foci (Z1-Z7) are delimited by vertical yellow lines. Nuclei in Z1 and Z2 are undergoing mitosis. They enter meiosis at Z3, which corresponds to the leptotene/zygotene stages (L/Z). Nuclei then proceed through pachytene (Z4-Z7). PMT, pre-meiotic tip; L/Z, leptotene/zygotene; EP, early pachytene; MP, mid-pachytene; LP, late pachytene. **(B)** Left, high-magnification image of a halfway-projection of a mid-pachytene nucleus co-stained with anti-HTP-3 (green), to trace chromosome axes, and anti-RAD-51 (magenta), to mark DSB repair sites. Chromosome selected for linearization is indicated by a yellow dashed line. Right, chromosome computationally linearized and divided into three equal length portions referred to as arms and center. Scale bar, 2 μm. **(C)** Histogram showing the distribution of RAD-51 foci at the center versus the arm regions of the chromosomes in different stages of meiosis in wild-type animals. Distribution of RAD-51 foci within the chromosome does not change among stages. Percentages are indicated above the bar graphs. A minimum of 50 chromosomes per condition from two independent biological repeats were linearized. Comparisons among different zones scored not significant by Fisher’s exact test, see S2 File. **(D)** Histogram showing the distribution of RAD-51 foci at the center versus the arms of the autosomes and X chromosomes in wild-type animals. Distribution of RAD-51 foci is similar between autosomes and the X chromosome. Percentages are indicated above the bar graphs. Comparisons among different zones scored not significant by Fisher’s exact test, see S2 File. **(E)** Graph depicting the relationship between IR dose in Gy (x-axis) and inferred mean number of DSBs (RAD-51 foci) per nucleus (y-axis). Predicted points based on [34] are in black, with their linear regression in black. Empirical data points (this work) are in green, with their linear regression in green. **(F)** Histogram shows the mean number of RAD-51 foci/nucleus (y-axis) scored along each zone in the germline (x-axis) exposed to the indicated IR doses in wild-type worms. At least 6 gonads were scored per IR dose. Error bars represent SEM for technical repeats from two biological repeats. Mean number of RAD-51 foci is indicated above each bar graph. All statistical comparisons are shown in S1 File. **(G)** Left, histogram shows the distribution of RAD-51 foci at the center versus the arm regions of the chromosomes in mid-pachytene for wild-type animals exposed to the indicated IR doses (x-axis). Distribution of RAD-51 foci within the chromosome is similar (most RAD-51 foci are present at the arms of the chromosomes) from 0 to 30 Gy; however, this distribution changes significantly to a more even distribution at 60 Gy and is enriched at the center of the chromosomes at 100 Gy. Percentages are indicated above the graphs. **p<0.01, ***p<0.001, ****p<0.001 by Fisher’s exact test. Right, representative images of linearized pachytene stage chromosomes from wild-type worms exposed to the indicated doses of IR, co-stained with anti-HTP-3 (green) to trace chromosome axes, and anti-RAD-51 (magenta) to mark DSB repair sites. Mean length (μm) of the linearized chromosomes is indicated to the right. A minimum of 50 chromosomes per condition were linearized. **(H)** Histogram depicts the mean number of RAD-51 foci/nucleus observed in different zones of the wild-type and *spo-11* mutant germlines + and–IR (60Gy). X-axis shows the position along the germline. At least 6 gonads were scored per IR dose. Error bars represent SEM for technical repeats from two biological repeats. Mean number of RAD-51 foci is indicated above each bar graph. ***p<0.001 by the two-tailed Mann-Whitney test, 95% C.I. Comparisons that scored significant between wild-type + 60 Gy and *spo-11* + 60 Gy are indicated. **(I)** Histogram shows the distribution of RAD-51 foci at the center versus the arm regions of the chromosomes in *spo-11* mutants compared with wild-type animals exposed to 60 Gy of IR. Percentages are indicated above each bar graph. Comparisons among different zones scored not significant by Fisher’s exact test, see S2 File.

Similar to COs, the distribution of SPO-11-dependent RAD-51 foci in mid-pachytene nuclei is biased towards the arms of the chromosomes during meiosis in wild type worms [8,11,27]. This suggests that early regulatory events may underlie the uneven distribution of DSB formation by SPO-11 and/or repair pathway choice. To assess whether DSB distribution is altered throughout pachytene progression (Fig 1A) we measured the localization of RAD-51 foci along the length of computationally straightened chromosomes in early, mid, and late pachytene. We did not score nuclei in the mid to late pachytene region (zone 6) to avoid scoring chromosomes in nuclei undergoing apoptosis, which have extremely high levels of RAD-51 staining [28,29].

Computationally straightened chromosomes were divided into three equal sized portions: two distal thirds, herein referred to as “arms”, flanking a central region (Fig 1B). We did not distinguish between the left and the right arms of the chromosomes, so all data for the arms is combined and RAD-51 foci are classified as being distributed either at the arms or the central region of the chromosomes. When RAD-51 foci overlapped or formed tracks, we used the diameter of a single focus (based on the mean diameter identified from multiple experiments) to determine the number of RAD-51 foci in those cases (S1A Fig). The biased distribution observed in mid-pachytene nuclei (82% of RAD-51 foci on the arms compared to 18% at the center, n = 50) was also detected for chromosomes in early and late pachytene nuclei (80% and 20% for both stages, n = 50 for each; Fig 1C), suggesting a biased distribution for endogenous DSBs from early to late stages of meiotic prophase I.

CO distribution is biased towards the chromosome arms, but this bias is less pronounced on the X chromosomes [9]. To examine if that might be due to RAD-51-marked DSBs being more evenly distributed on the X chromosomes, we identified the X by staining dissected gonads with a pan histone acetylation antibody which is detected on autosomes but is depleted on the X chromosome during pachytene (S1B Fig) [24]. Interestingly, the X chromosome exhibited a similar off-center enriched distribution of RAD-51 foci compared to autosomes at all measured stages (Fig 1D; p = 0.6176 at mid-pachytene by the Fisher’s exact test).

Studies in various organisms showed that exogenous DSBs can be introduced during meiosis to produce recombination products similarly to endogenous SPO-11-dependent DSBs [30–33]. Moreover, we previously showed that 60 Gy of gamma-irradiation eliminates the arm-biased distribution of DSBs during mid-pachytene in wild type, resulting in an even distribution of RAD-51 along the chromosomes [27]. However, the threshold level of DSBs required to eliminate this biased distribution remains to be determined. Therefore, to identify a dose that would produce equal levels of RAD-51 foci at the center and the arms of the bivalents we irradiated worms with 10, 20, 30, 60 and 100 Gy (S1C Fig). It has been proposed that a dose of 10 Gy results in ~3.9 DSBs/chromosome in *C*. *elegans* [34]. Since *C*. *elegans* hermaphrodites have 6 pairs of chromosomes per nucleus, we would expect to see an average of 23.4 DSBs/nucleus at that dose. Instead, we observed a maximum of 11.8 RAD-51 foci in mid-pachytene nuclei (Fig 1E and 1F). Moreover, mitotic nuclei in the premeiotic tip (zones 1 and 2) and meiotic nuclei in late pachytene (zone 7) exhibited lower levels of RAD-51 foci compared to nuclei in leptotene to mid-pachytene stages at every dose (Fig 1F). These results suggest that either lower levels of exogenous DSBs are produced than previously hypothesized or the processing of exogenous DSBs occurs with faster kinetics and/or via alternative pathways to HR during mitosis and late pachytene.

The off-center distribution of RAD-51 foci was progressively reduced with higher doses of irradiation (Fisher’s exact test, p≥0.1198 for all stages at 10, 20 and 30 Gy; Figs 1G and S1D), reaching a nearly homogenous distribution at 60 Gy (mid-pachytene, p ≤ 0.0104; Fig 1G and [27]). Surprisingly, a significant enrichment for RAD-51 foci was observed at the center of the chromosomes at 100 Gy (50% for both arms combined vs 50% at the center; p<0.0001 by the Fisher’s exact test for all stages compared to 0 Gy and p≤0.0004 for all stages compared to 60 Gy, Figs 1G and S1D). To distinguish between the effects of exogenous DSBs alone compared with both endogenous and exogenous breaks, we quantified RAD-51 foci following a 60 Gy exposure in germline nuclei from *spo-11* and *dsb-1* single mutants, in which endogenous meiotic DSBs are abrogated [31,35]. Exclusive formation of exogenous DSBs in both mutant backgrounds resulted in a mean number of RAD-51 foci per nucleus comparable to wild type worms exposed to 60 Gy at every region of the germline (Figs 1H and S1E). Higher mean levels of RAD-51 foci per nucleus were observed in late pachytene in *spo-11* (zones 6 and 7, p<0.0001 by the Mann Whitney U-test) and *dsb-1* worms (zone 6; p = 0.002), probably corresponding to nuclei undergoing apoptosis. Both *spo-11* and *dsb-1* mutants exposed to irradiation exhibited an even distribution of RAD-51 foci between arms and center regions similar to wild type worms (Figs 1I and S1F). Altogether, these data suggest that RAD-51-marked DSB repair sites are biased towards the arms of the X chromosome and the autosomes at all stages of pachytene, and that introducing exogenous DSBs abrogates this biased distribution.

### Blocking RAD-51 nucleofilament turnover mimics the unbiased distribution achieved via exogenous DSB formation

**Fig 2:**
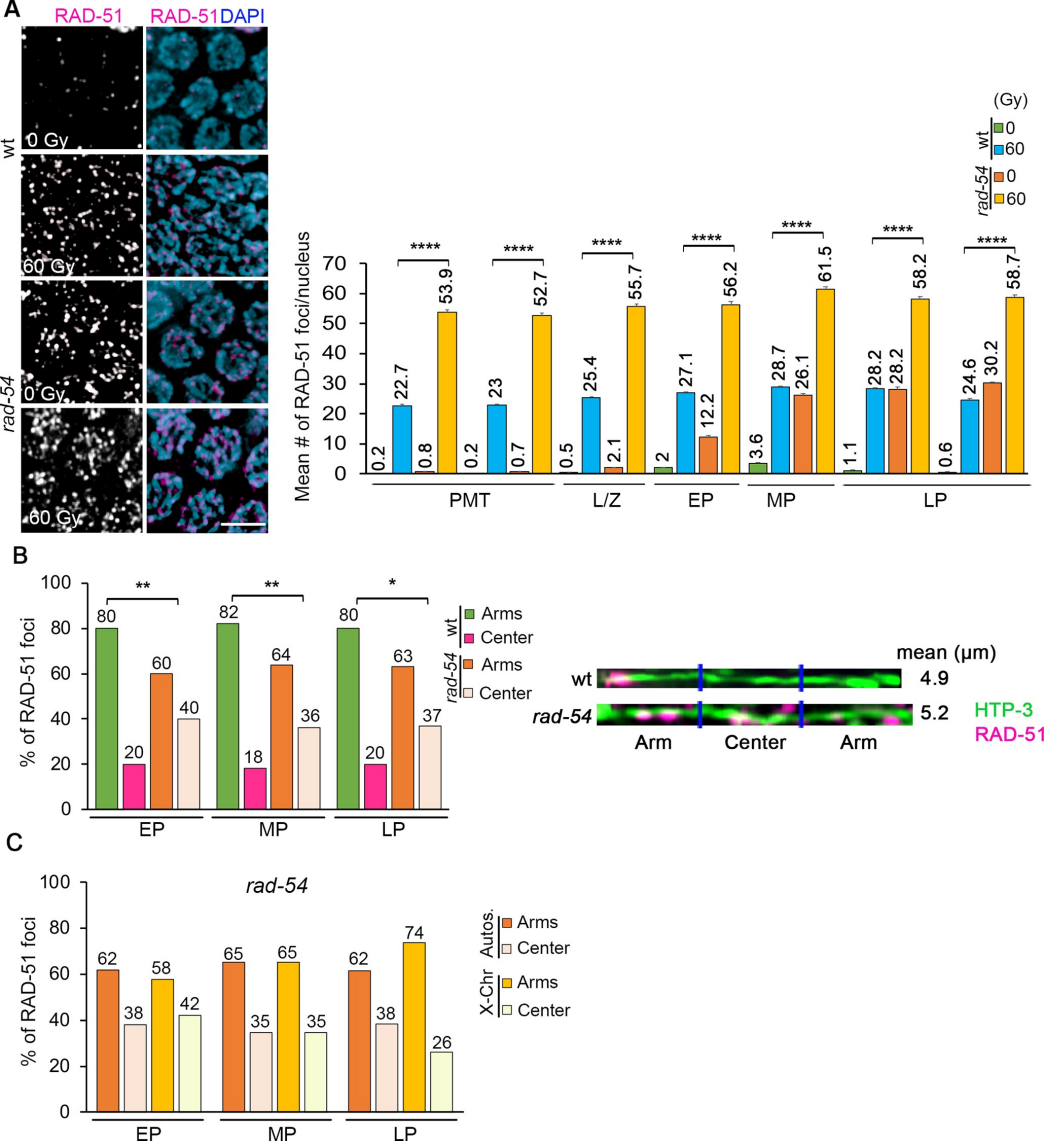
Blocked RAD-51 nucleofilament turnover results in unbiased distribution. **(A)** Left, high-resolution images of mid-pachytene nuclei (zone 5) immunostained for RAD-51 (magenta) and co-stained with DAPI (blue). Scale bar, 2 μm. Right, histogram shows the mean number of RAD-51 foci/nucleus (y-axis) scored along each zone in the germline (x-axis) of the indicated genotypes and conditions. At least 6 gonads were scored per genotype. Mean number of RAD-51 foci is indicated above each bar graph. Error bars represent SEM from technical repeats for each of two biological replicates. ****p<0.0001. Comparisons that scored significant between wild-type + 60 Gy and *rad-54* + 60 Gy are indicated. **(B)** Left, histogram shows the distribution of RAD-51 foci at the center versus the arms of the chromosomes throughout pachytene (early to late) in *rad-54* mutants compared to wild-type animals + and–IR (60 Gy). RAD-51 foci are more evenly distributed throughout the chromosomes in *rad-54* mutants compared to wild-type. Percentages are indicated above each bar graph. *p<0.05, **p<0.01. Right, representative images of linearized chromosomes from *rad-54* and wild-type worms co-stained with anti-HTP-3 (green), and anti-RAD-51 (magenta). A minimum of 50 chromosomes per condition were linearized. Mean length (μm) of the linearized chromosomes is indicated to the right. **(C)** Histogram shows the distribution of RAD-51 foci at the center versus the arm regions of the autosomes and X chromosomes in mid-pachytene stage in *rad-54* mutants. Percentages are indicated above each bar graph. Comparisons among different zones scored not significant by Fisher’s exact test, see S2 File.

Following DSB end resection, RAD-51 loads on the 3’-ssDNA ends forming a nucleoprotein filament required for strand invasion/exchange, but after strand exchange it must dissociate from the DNA to allow for completion of recombination [15]. To determine if blocking RAD-51 nucleofilament turnover results in an even distribution pattern between the arms and center of the chromosomes throughout pachytene, we analyzed *rad-54* mutants. Loss of RAD-54 protein perturbs the progression of HR by blocking the turnover of RAD-51 nucleofilaments, therefore trapping RAD-51 foci at all DSB repair sites ([36]; Fig 2A). Analysis of RAD-51 foci showed a homogeneous distribution of DSBs between arms and center regions of the chromosomes from early to late pachytene in *rad-54* mutants (Fig 2B; p = 0.5844, and [37]). This is significantly different from the off-centered distribution of RAD-51 foci observed in wild type (p = 0.0031 EP, p = 0.0039 MP, p = 0.0148 LP). Moreover, we observed an even distribution of RAD-51-marked breaks between arms and center both for the autosomes and the X chromosome throughout pachytene (Fig 2C, p = 0.8176 and p = 0.2444, respectively). We measured an average of 3.34 RAD-51 foci/chromosome in mid-pachytene nuclei, 2.9 on the X chromosomes and 3.8 on the autosomes (n = 24 and 26, respectively, p<0.0001), consistent with our previous data [24]. To determine whether additional DSB formation could alter the homogeneous distribution observed in *rad-54* mutants these were exposed to gamma-irradiation (IR). *rad-54* mutants dosed with 60 Gy exhibited elevated levels of RAD-51 foci in all zones compared to wild type + IR (Fig 2A) and continued to show an unbiased RAD-51 foci distribution along the chromosomes (Fig S2A; p = 0.3199 EP, p = 0.6419 MP, and p = 0.9008 LP). Taken together, these results suggest that blocking RAD-51 nucleofilament turnover results in unbiased DSB distribution throughout pachytene on both autosomes and the X chromosome.

### Condensin function but not total axis length affects RAD-51 distribution on chromosomes

**Fig 3:**
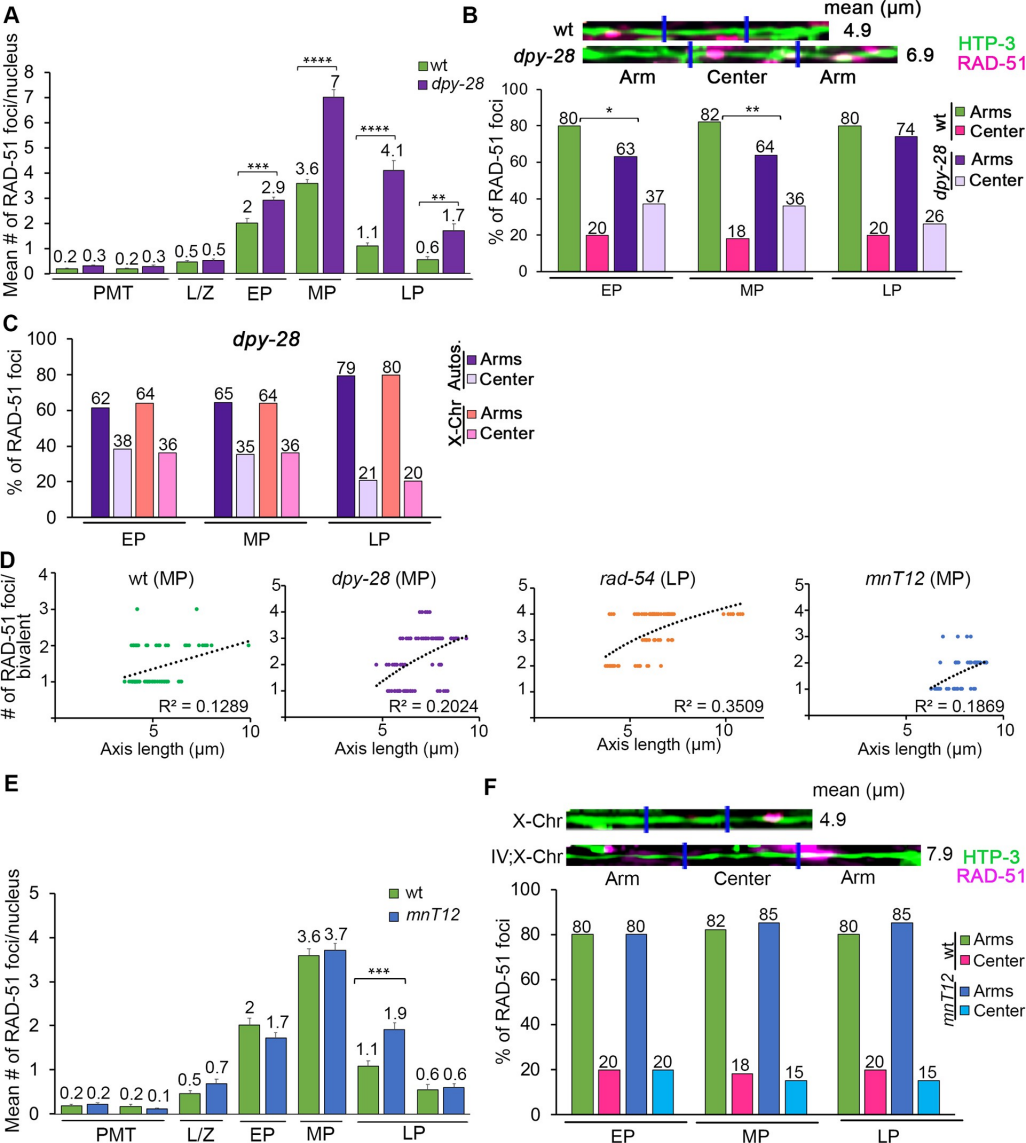
Condensin function, but not total chromosome axis length, regulates the distribution of RAD-51 foci along the chromosomes. **(A)** Histogram shows the mean number of RAD-51 foci/nucleus (y-axis) scored along each zone in the germlines (x-axis) of *dpy-28* mutants compared to wild-type. *dpy-28* mutants exhibit a significant increase in the number of RAD-51 foci during pachytene (EP to LP). At least 6 gonads were scored per genotype. Mean number of RAD-51 foci is indicated above each bar graph. Error bars represent SEM from technical repeats for each of two biological replicates. **p<0.01, ***p<0.001, ****p<0.0001 by the two-tailed Mann-Whitney test, 95% C.I. **(B)** Top shows representative images of linearized chromosomes co-stained with anti-HTP-3 (green) and anti-RAD-51 (magenta). Mean length (μm) of the linearized chromosomes is indicated to the right. Bottom, histogram shows the distribution of RAD-51 foci at the center versus the arms of the chromosomes in *dpy-28* mutants compared with wild-type. The distribution of RAD-51 foci in *dpy-28* mutants is significantly different from wild-type in early and mid-pachytene stages. *p<0.05, **p<0.01 by Fisher’s exact test. **(C)** Histogram shows the distribution of RAD-51 foci at the center versus the arms of the chromosomes throughout pachytene in *dpy-28* mutants comparing autosomes and X chromosomes. Percentages are indicated above each bar graph. Comparisons among different zones scored not significant by Fisher’s exact test, see S2 File. **(D)** Graphs depicting the relationship between the axis length (μm) and the number of RAD-51 foci in the indicated genotypes and stages. Linear regression (black dotted line) and R^2^ value are indicated. **(E)** Histogram shows the mean number of RAD-51 foci/nucleus (y-axis) scored along each zone in the germlines (x-axis) of the *mnT12* mutants compared to wild-type. *mnT12* mutants exhibit a similar number of RAD-51 foci compared to wild-type except for a significant increase in late pachytene stage. At least 6 gonads were scored per genotype. Mean number of RAD-51 foci is indicated above the bar graphs. Error bars represent SEM from technical repeats for each of two biological replicates. ***p<0.001, by the two-tailed Mann-Whitney test, 95% C.I. **(F)** Top shows representative images of linearized chromosomes co-stained with anti-HTP-3 (green) and anti-RAD-51 (magenta) from *mnT12* mutant (IV;X-Chr) and wild-type worms (X-Chr). Mean length (μm) of linearized chromosomes is indicated to the right. Bottom, histogram shows a similar distribution of RAD-51 foci at the center versus the arms of the chromosomes in *mnT12* mutants compared to wild-type throughout pachytene. Comparisons among different zones scored not significant by Fisher’s exact test, see S2 File.

To assess the effect of chromosome axis length on the regulation of RAD-51 foci distribution, we first examined a *dpy-28* mutant that lacks a subunit of the condensin complex. In *dpy-28* mutants chromosome axes are extended, both the levels and distribution of meiotic DSBs and CO recombination along the X chromosome are altered [36], and levels of RAD-51 foci are elevated throughout pachytene on all chromosomes (Fig 3A). In early and mid-pachytene stages, RAD-51 foci were more evenly distributed between the arm and center regions in *dpy-28* compared to wild type (Fig 3B, p = 0.0103 and 0.0018, respectively, by the Fisher’s exact test). By late pachytene this even distribution was no longer observed (p = 0.3936), suggesting condensin function may primarily play role in regulating RAD-51 distribution in early and mid-pachytene. This pattern, consisting of an even distribution in early and mid-pachytene and a biased distribution in late pachytene, was observed both on the autosomes and the X chromosomes in *dpy-28* mutants (Fig 3C). To determine if the biased distribution persisting in late pachytene might be linked to DSB levels, we assessed *dpy-28* mutants treated with a dose of 60 Gy. Irradiation significantly increased the number of breaks during leptotene/zygotene through late pachytene in *dpy-28* gonads compared to wild type (S2B Fig; p<0.0001 Zones 3–6, p = 0.003 Zone 7, Mann Whitney U-test). Moreover, an unbiased distribution of RAD-51 foci was observed after IR treatment in mid and late pachytene (S2C Fig; p = 0.3185 Zone 5, p = 0.7951 Zone 7 compared to wild type + IR).

The average length of chromosome axes in mid-pachytene nuclei was increased in *dpy-28* compared to wild type (Fig 3B and 3D and S2 File, 6.94μm and 4.97μm, n = 76 and n = 130, respectively; p<0.0001 by the Student t-test). Moreover, the average total axis length was shorter for the X chromosome compared to the autosomes (4.26μm and 5.39μm, n = 49 and 81 respectively in mid-pachytene, p<0.0001), and in the absence of DPY-28 a 20% reduction in length was observed for the X chromosome relative to the autosomes in mid-pachytene nuclei (6.18μm and 8.09μm, n = 46 and n = 36 respectively; p<0.0001), similar to what was previously reported for chromosome I and the X chromosome in wild type and *dpy-28* backgrounds [36]. Altered axis length in *dpy-28* mutants may differentially affect the X chromosomes since lower levels of RAD-51 foci were observed along the X compared to the autosomes in mid-pachytene (a mean of 2.04 and 2.53 RAD-51 foci, n = 46 and n = 36, respectively; p = 0.0218, Mann Whitney U-test; S2 File).

Elevated doses of gamma irradiation also increased chromosome axis length. Wild type worms irradiated with 60 Gy exhibited an increase, albeit not significant, in chromosome axis length compared to unirradiated wild type control (5.11 μm and 4.97 μm at mid-pachytene, n = 80 and n = 130, respectively; p = 0.31, t-test), whereas a dose of 100 Gy resulted in a significant increase in average total axis length (6.22 μm and 4.97 μm, n = 50 and n = 130, respectively; p<0.0001; S2 File). This increase in axis length at 100 Gy could contribute to the observed enrichment of RAD-51 foci at the center of the chromosomes (Fig 1G).

We observed a slight positive correlation between chromosome axis length and the number of breaks per chromosome (Fig 3D, wild type R^2^ = 0.13 and *dpy-28* R^2^ = 0.20 in mid-pachytene, following a linear and logarithmic trendline, respectively). A stronger correlation was observed for late pachytene chromosomes in *rad-54* mutants (Fig 3D). However, axis length alone does not fully explain RAD-51 distribution, since the regression coefficient of determination is low (*rad-54* late pachytene R^2^ = 0.35, following a logarithmic trendline).

To determine whether the loss in biased RAD-51 distribution is also observed when chromosome axis length is increased independent of a genetic mutation, we examined *mnT12* worms that carry a stable homozygous IV:X chromosome fusion which nearly doubles the axis length compared to wild type (4.26 ± 0.07 μm and 7.86 ± 0.11 μm for the X and IV:X fusion, respectively; [38,39] and this study). *mnT12* worms exhibited RAD-51 foci levels and a biased off-center distribution of RAD-51 foci, that were similar to wild type (Fig 3E-3F). A 60 Gy exposure resulted in similar levels of RAD-51 foci in *mnT12* worms compared to wild type (S2D Fig), and an even distribution of RAD-51 foci along the length of the chromosomes throughout pachytene (S2E Fig). However, the regression coefficient of determination for the number of breaks against axial length in unirradiated *mnT12* worms is low (Fig 3D, R^2^ = 0.19, following a logarithmic trendline, at mid-pachytene). The fact that a chromosome fusion exhibits a biased distribution of RAD-51 foci towards the arms suggests that the center domain is not determined by DNA sequence but by physical position. Our analysis also shows that the number of breaks per chromosome is not altered relative to the total chromosome axis length. Collectively, this suggests that alterations in condensin function, but not total chromosome axis length, can influence RAD-51 distribution.

### The chromatin landscape at the arms/center affects RAD-51 foci distribution

**Fig 4:**
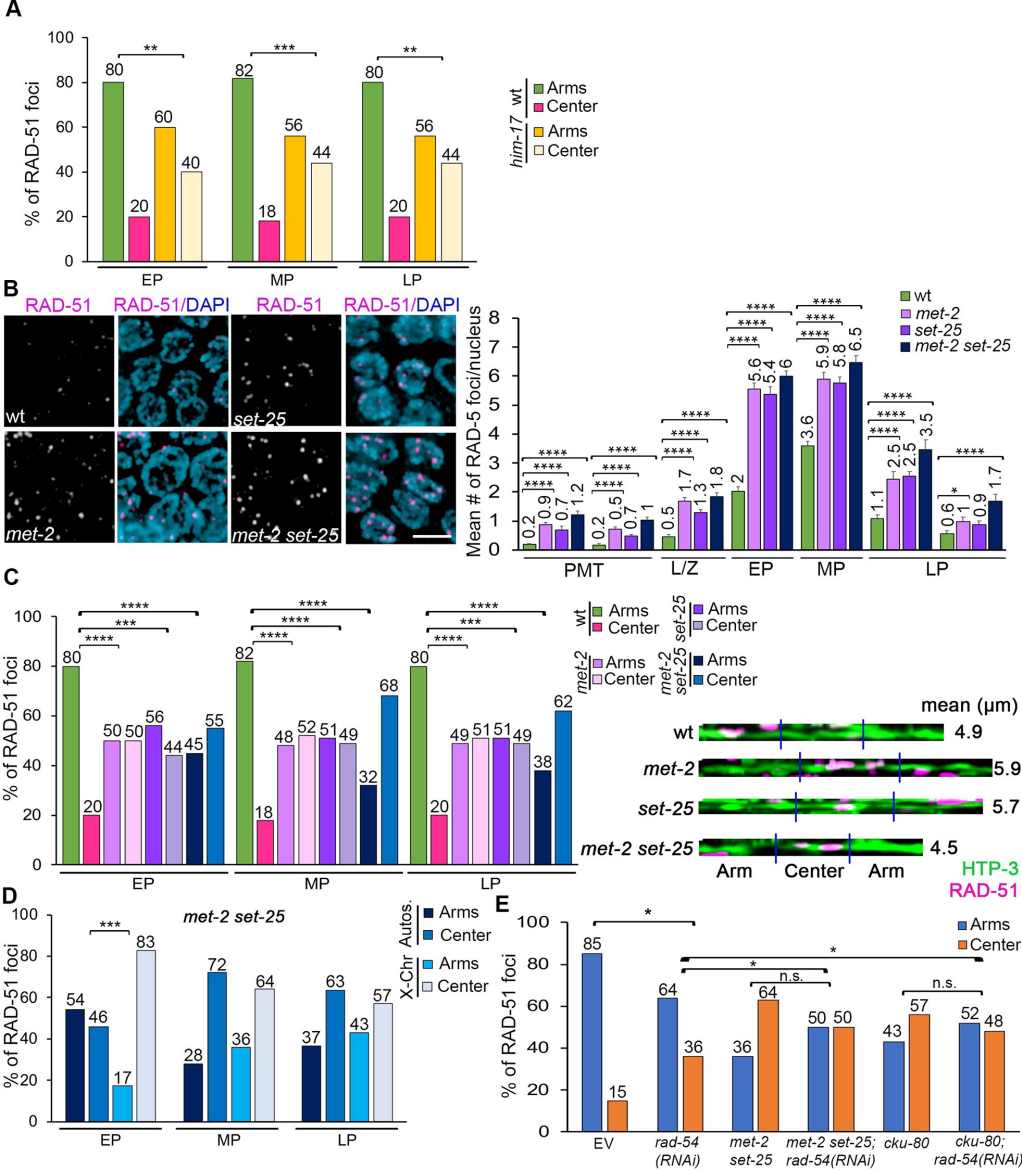
The chromatin landscape at the arms/center influences RAD-51 foci distribution. **(A)** Histogram shows the distribution of RAD-51 foci at the center versus the arms of the chromosomes throughout pachytene (early to late) in *him-17* mutants compared to wild-type. *him-17* mutants show an even distribution of RAD-51 foci between arms and center compared to wild-type worms. Percentages are indicated above each bar graph. **p<0.01, ***p<0.001 by Fisher’s exact test. **(B)** Left, high-resolution images of mid-pachytene nuclei (zone 5) immunostained for RAD-51 (magenta) and co-stained with DAPI (blue). Scale bar, 2 μm. Right, histogram shows the mean number of RAD-51 foci/nucleus (y-axis) scored along each zone in the germlines (x-axis) of the indicated genotypes. At least 6 gonads were scored per genotype. Mean number of RAD-51 foci is indicated above each bar graph. Error bars represent SEM from technical repeats for each of two biological replicates. *p<0.05, ****p<0.0001 by the two-tailed Mann-Whitney test, 95% C.I. **(C)** Left, histogram shows the distribution of RAD-51 foci at the center versus the arms of the chromosomes throughout pachytene in *met-2*, *set-25*, and *met-2 set-25* mutants compared to wild-type. The distribution of RAD-51 foci in *met-2*, *set-25*, and *met-2 set-25* mutants is significantly different compared to wild-type. Percentages are indicated above each bar graph. Right, representative images of linearized chromosomes of the indicated genotypes co-stained with anti-HTP-3 (green) and anti-RAD-51 (magenta). Mean length (μm) of the linearized chromosomes is indicated to the right. A minimum of 50 chromosomes per condition were linearized. **(D)** Histogram shows the distribution of RAD-51 foci at the center versus the arms of the chromosomes throughout pachytene in *met-2 set-25* double mutants comparing autosomes and X chromosomes). Percentages are indicated above each bar graph. Distribution of RAD-51 foci is significantly different between autosomes and X chromosomes in early pachytene stage. ***p<0.001 by Fisher’s exact test. **(E)** Histogram shows the distribution of RAD-51 foci at the center versus the arms of the chromosomes in the indicated genotypes. Percentages are indicated above each bar graph. Distribution of RAD-51 foci is biased towards the center of the chromosome and significantly different in *met-2 set-25;rad-54(RNAi)* and *cku-80;rad-54(RNAi)* when compared to *rad-54(RNAi)* but not compared to *met-2 set-25;EV* or *cku-80;EV*, respectively. *p<0.05 by Fisher’s exact test. n.s., not significant.

To further explore how chromatin accessibility/compaction affects localization of RAD-51 foci preferentially to the arm regions of the chromosomes, we examined mutants for genes that regulate histone methylation. Histone H3 dimethylation on lysine 9 (H3K9me2) is enriched on the arms of the chromosomes [40,41] and HIM-17, a protein required for H3K9me2 in *C*. *elegans* [19], regulates the distribution of RAD-51 foci towards chromosome arms in mid-pachytene [27]. Extending these earlier findings, we observed a similar enrichment of RAD-51 foci at the center region of the chromosomes in *him-17* mutants compared to wild type in early and late pachytene compared to mid-pachytene (Fig 4A; p = 0.0013 EP, p = 0.0004 MP, p = 0.0021 LP). Moreover, there was a stronger center-enrichment effect on the X chromosome compared to the autosomes in early and mid-pachytene, whereas RAD-51 foci are more evenly distributed along autosomes at those stages (S3A Fig). Irradiating *him-17* worms with 60 Gy resulted in higher number of RAD-51 foci per nucleus in late pachytene compared to wild type (S3B Fig), and an even distribution of RAD-51 between arms and center regions throughout pachytene, similar to IR-treated wild type worms (S3C Fig). These results link H3K9me2 with the regulation of RAD-51 foci distribution in the distal thirds of the chromosomes.

Given that HIM-17 has multiple roles in meiosis and how it regulates H3K9me2 remains unknown, we also assessed single and double mutants for *met-2* and *set-25* which encode for two well-characterized histone methyltransferases functioning in the germline. MET-2 is responsible for H3K9 mono and dimethylation, and SET-25 regulates H3K9me3 that is enriched on the arms compared to the center of the chromosomes [40–43]. *met-2* and *set-25* single and double mutants exhibited higher levels of RAD-51 foci compared to wild type in nuclei at the premeiotic tip, as previously reported [41], as well as in leptotene/zygotene, early, mid and late pachytene (Fig 4B). *met-2* and *set-25* single mutants showed a biased RAD-51 distribution towards the center of the chromosomes compared to wild type, and this was further exacerbated in the *met-2 set-25* double mutant (Fig 4C; p-value between <0.0001 and 0.0008, for all zones, Fisher’s exact test). Moreover, RAD-51 foci distribution was similar between the X chromosome and the autosomes at all pachytene stages, except in early pachytene for *set-25* (S3A Fig, p = 0.0433) and *met-2 set-25* (Fig 4D, p = 0.0005). IR treatment (60 Gy) of *met-2* and *met-2 set-25* resulted in increased levels of RAD-51 foci in mid and late pachytene, whereas *set-25* mutants showed similar levels to wild type + 60 Gy (S3D Fig). Similar to *him-17*, IR produced an even distribution of RAD-51 foci between the arms and the center in *met-2* and *set-25* single and double mutants (S3C Fig). In contrast, RAD-51 foci remained enriched at the center of the chromosomes upon RAD-54 depletion by RNAi in *met-2 set-25* double mutants (Fig 4E, p = 0.1290 when comparing *met-2 set-25;rad-54(RNAi)* and *met-2 set-25* treated with an empty vector), in both autosomes and the X chromosome (S3E Fig). Taken together, these data indicate that lack of H3K9me2/3 regulation alters the distribution of RAD-51 foci.

### DSB recognition cKU-70/80 heterodimer contributes to the off-center distribution of RAD-51-marked DSB repair sites

**Fig 5:**
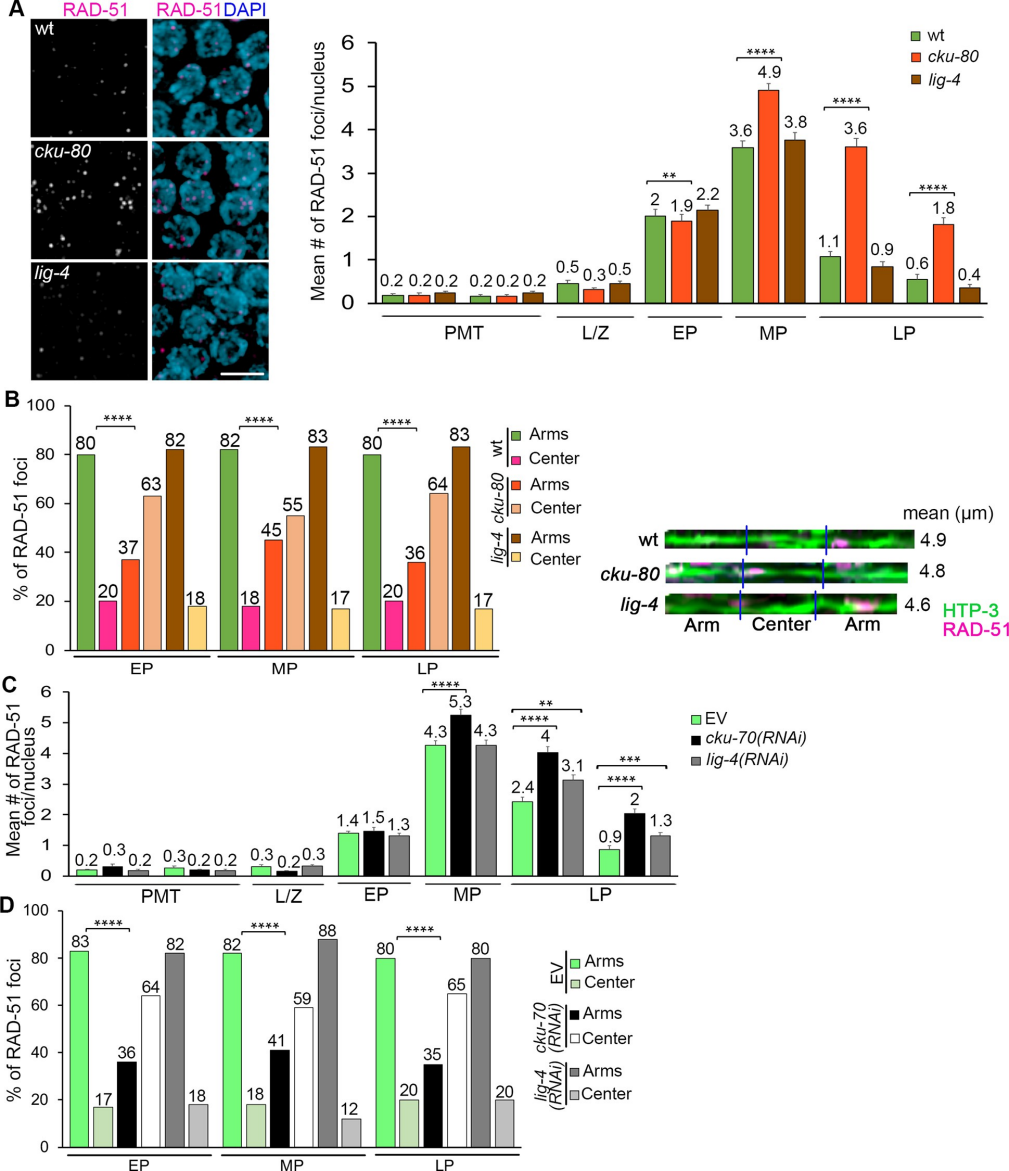
cKU-70/80 contributes to the biased distribution of RAD-51 foci along the chromosome. **(A)** Left, high-resolution images of mid-pachytene nuclei (zone 5) immunostained for RAD-51 (magenta) and co-stained with DAPI (blue) from the indicated genotypes. Scale bar, 2 μm. Right, histogram shows the mean number of RAD-51 foci/nucleus (y-axis) scored along each zone in the germlines (x-axis) of the indicated genotypes. *cku-80* mutant animals exhibit an increase in the number of RAD-51 foci throughout pachytene compared to wild-type worms, whereas *lig-4* mutants show numbers of RAD-51 foci similar to wild-type. At least 6 gonads were scored per genotype. Mean number of RAD-51 foci is indicated above each bar graph. Error bars represent SEM from technical repeats for each of two biological replicates. **p<0.01, ****p<0.0001 by the two-tailed Mann-Whitney test, 95% C.I. **(B)** Left, histogram shows the distribution of RAD-51 foci at the center versus the arm regions of the chromosomes throughout pachytene in *cku-80* and *lig-4* mutants compared to wild-type. *cku80* mutants exhibit a more even distribution of RAD-51 foci compared to wild-type. Percentages are indicated above each bar graph. Right, representative images of linearized chromosomes for the indicated genotypes co-stained with anti-HTP-3 (green) and anti-RAD-51 (magenta). Mean length (μm) of the linearized chromosomes is indicated to the right. A minimum of 50 chromosomes per condition were linearized. ****p<0.0001 by Fisher’s exact test. **(C)** Histogram shows the mean number of RAD-51 foci/nucleus (y-axis) scored along each zone in the germlines (x-axis) following RNAi depletion. *cku-70(RNAi)* animals show a significant increase in the number of RAD-51 foci compared to empty vector (EV) wild-type worms starting at mid-pachytene. At least 6 gonads were scored per genotype. Mean number is indicated above each bar graph. Error bars represent SEM from technical repeats for each of two biological replicates. **p<0.01, ***p<0.001, ****p<0.0001 by the two-tailed Mann-Whitney test, 95% C.I. **(D)** Histogram shows the distribution of RAD-51 foci at the center versus the arms of the chromosomes throughout pachytene in *cku-70(RNAi)* and *lig-4(RNAi)* worms compared to wild-type animals (EV). Distribution of RAD-51 foci along the chromosomes in *cku70(RNAi)* animals is more even than in wild-type worms. Percentages are indicated above each bar graph. ****p<0.0001 by Fisher’s exact test.

The cKU-70/80 heterodimer is generally involved in channeling DSB repair towards NHEJ [44], but it has also been implicated in DSB end protection, the inhibition of alternative end-joining pathways, and the extension of the resection track in replication stress single-ended DSBs [45]. We therefore examined *cku-70/80* mutants to determine whether the distribution of RAD-51-marked DSB repair sites could be regulated at the level of the DSB repair machinery that is recruited to different regions of the chromosomes. *cku-80* mutants exhibited higher levels of RAD-51 foci in mid to late pachytene nuclei compared to wild type (Fig 5A; p = 0.0012 Zone 4, p<0.0001 Zones 5–7), whereas exposure to 60 Gy resulted in elevated RAD-51 foci levels in the premeiotic tip and late pachytene compared to wild type (S4A Fig; p = 0.0008 Zone 1, p = 0.0004 Zone 2, p<0.0001 Zones 6 and 7), consistent with a role for NHEJ in mitosis and late prophase I DSB repair [25,26,46]. *cku-80* mutants exhibited a distinct distribution of RAD-51 foci compared to wild type, with a significant enrichment at the center rather than at the arms of the chromosomes (Fig 5B; for example, in mid pachytene, p<0.0001, Fisher’s exact test), in both autosomes and the X chromosome throughout pachytene (S4B Fig, p≥0.6334). Exogenous DSBs (60 Gy) produced a homogeneous distribution of RAD-51 foci between arms and center regions on both autosomes and the X chromosomes (S4B and S4C Fig). In contrast, the enrichment of RAD-51 foci at the center of the chromosomes persisted upon RAD-54 depletion by RNAi in *cku-80* mutants (Fig 4E, p = 0.3185 comparing *cku-80;rad-54(RNAi)* and *cku-80;EV*), in both autosomes and the X chromosome (S3E Fig). Moreover, analysis of the distribution of the CO promoting factor ZHP-3/RNF212/Zip3 showed that while COs are biased to an off-center position in wild type (S4D Fig, 96% of ZHP-3 foci at the arms and 4% at the center), levels of ZHP-3 foci at the center of the chromosomes were significantly increased in *cku-80* worms (29%, p = 0.0007). This analysis indicates that RAD-51 foci enriched at the center third of chromosomes can be shepherded down a CO-associated repair pathway. To distinguish whether the enrichment of RAD-51 foci at the center in *cku-80* mutants was a result of losing the early recognition of breaks by cKU-80 or due to the NHEJ pathway influencing break distribution, we examined *lig-4* mutants that lack the ligase that rejoins DNA ends in the canonical NHEJ pathway [44,47]. In contrast to *cku-80* mutants, lack of LIG-4 did not result in higher levels of RAD-51 foci in mid or late pachytene nuclei (Fig 5A, p ≥0.0961 for all zones), and higher levels of RAD-51 foci were only observed after a 60 Gy exposure (S4A Fig, p<0.001 in all zones except mid-pachytene where p = 0.55). Similar to wild type, the distribution of RAD-51 foci along the length of the chromosomes was biased to the arms in *lig-4* mutants (Fig 5B, p≥0.4110 for all zones), and unbiased after IR in both autosomes and the X chromosome (S4B and S4C Fig). Analysis of *cku-70(RNAi)* and *lig-4(RNAi)* worms replicated the patterns and levels observed in *cku-80* and *lig-4* mutants ruling out allele-specific effects (Figs 5C and 5D and S5). Altogether, these results suggest that recognition of DSBs by the cKU-70/80 heterodimer, but not their activity promoting NHEJ repair, can influence the loading of RAD-51 and/or the processing of DSBs at different regions of the chromosomes during meiosis.

## Discussion

**Fig 6:**
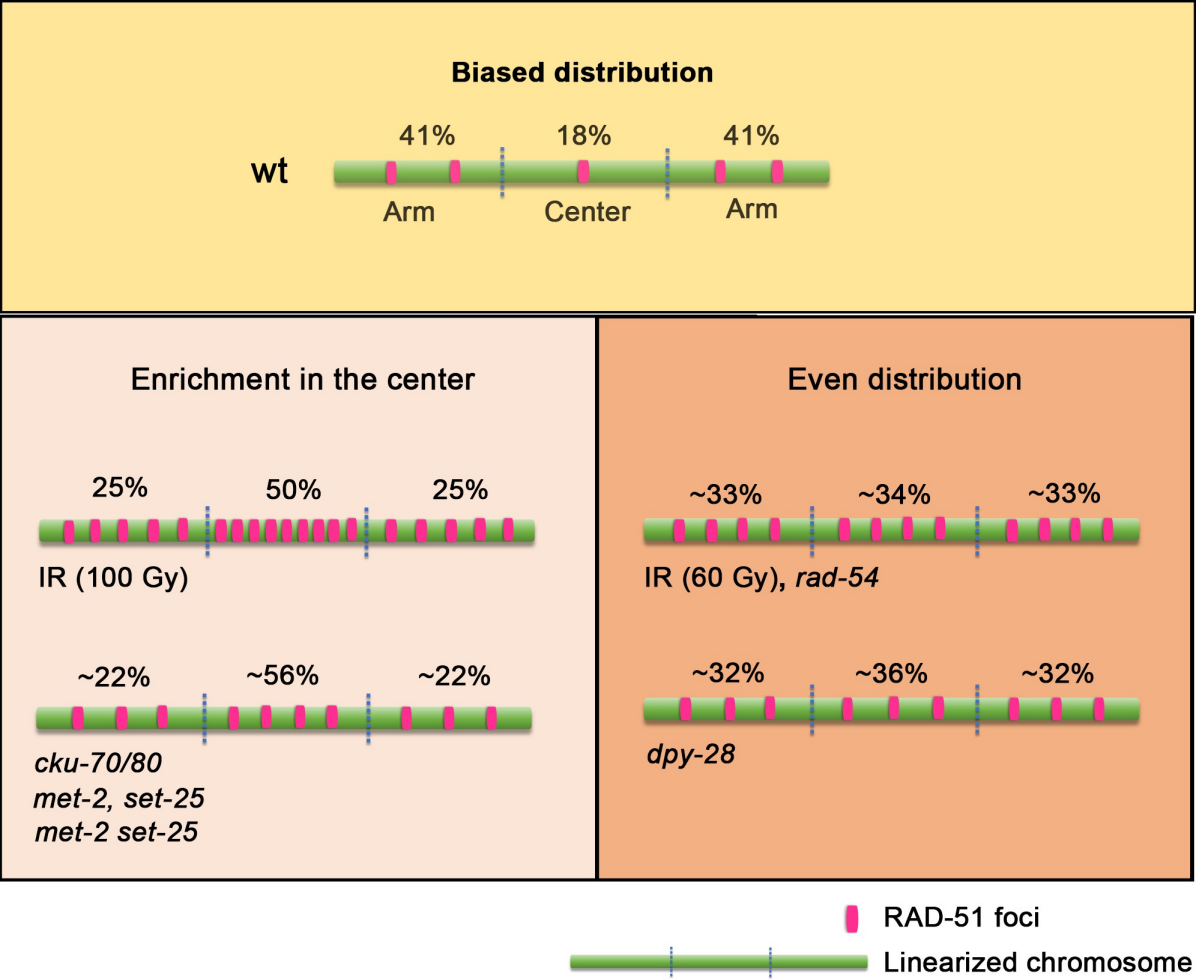
Illustration showing how different factors contribute to the regulation of meiotic RAD-51 foci distribution. Under normal conditions the distribution of RAD-51 foci is biased to the arms of the chromosomes throughout pachytene in wild-type animals. Our analysis identifies various factors contributing to the regulation of meiotic DSB patterning. An enrichment of RAD-51 foci is detected at the center of the chromosomes following exposure to very high doses of exogenous DSBs (100 Gy), when DSB recognition by cKU-70/80 is impaired, and when the chromatin landscape is perturbed (*met-2*, *set-25*, *met-2 set-25*). An even distribution of RAD-51 foci throughout the arms and the center of the chromosomes is observed upon intermediate doses of exogenous DSBs (60 Gy), when RAD-51 nucleofilament turnover is blocked (*rad-54*), and when chromosome compaction (*dpy-28*) is altered. An average of the percentages of RAD-51 foci observed at the arms or the center during mid-pachytene are shown above each chromosome. Note that the percentages above each of the arms are arbitrarily assigned since we cannot make a distinction between right or left arms. Chromosome axes are indicated in green and RAD-51 foci in magenta.

CO frequency is tightly regulated in *C*. *elegans*, resulting in a single CO between each pair of homologous chromosomes [48]. Among self-fertilizing organisms, worms have very low levels of genetic polymorphisms and high linkage disequilibrium [49,9]. Low levels of CO recombination, preferentially in gene-depleted regions, may be important to ensure certain combinations of alleles always work together [17,40]. Although some *C*. *elegans* mutants and changes in temperature have been described to shift CO recombination events towards the center of the chromosomes [20,21,37,50–53] it seemed that this bias was produced at the CO designation level since DSB formation was reported to be homogeneous in a *rad-54* background [37]. In the present study, we show that DSB repair sites marked by RAD-51 foci in wild type are biased towards the distal thirds of the chromosomes throughout pachytene, and that this is controlled by the chromatin environment and genetic factors (Fig 6).

Our results indicate a biased distribution of RAD-51 foci towards the distal arms of the chromosomes throughout pachytene progression, since we observed an 80% enrichment at the arms of the chromosomes (instead of the expected 66%) in early, mid, and late pachytene in wild type (Fig 1C). Our data implies that processing of DSBs into COs is biased to the arms of the bivalents throughout pachytene. The only examples of a different distribution for RAD-51-marked DSB repair sites throughout pachytene were *dpy-28* and *met-2 set-25* when comparing the autosomes to the X chromosome (Figs 3C and 4D, respectively). Alterations in the compaction or configuration of the chromatin in these backgrounds could partly explain why the position of DSB processing sites along the chromosome is altered throughout pachytene progression, especially in the X chromosome because it has different chromatin marks and transcription is blocked until very late pachytene compared to the autosomes [54,55]. Sex chromosomes are regulated differently in many organisms, particularly in the heterogametic sex [56–59]. Since the off-center enrichment in CO distribution observed in autosomes is more pronounced than on the X [9,18], one would expect the distribution of RAD-51 foci to be less biased towards the arms on the X chromosome. However, in general we observed a similar distribution of RAD-51 foci for both the X and the autosomes.

### A homogeneous distribution of RAD-51 foci occurs following an excess of DSBs

The enrichment of RAD-51 foci on the arms could be due to biased processing of DSBs on those regions to promote a distinct recombination outcome. Using gamma irradiation, breaks are potentially formed randomly and simultaneously along the length of the chromosomes (Fig 6). However, we still observed a bias in the distribution of RAD-51 foci towards the arms at doses up to 30 Gy, with an average of 25.5 RAD-51 foci/nucleus in mid-pachytene and approximately 4.25 per chromosome (Fig 1F). This suggests that if IR-induced breaks are evenly distributed along the chromosomes, they are processed differently at the arms than at the center of the chromosomes. Only 60 Gy produced a homogeneous distribution of RAD-51 foci along the length of the chromosomes. An important observation from this study is that the levels of RAD-51 foci detected at higher doses of irradiation are lower than those predicted based on the number of COSA-1 CO-designated sites observed after low doses of irradiation (1, 2.5 and 5 Gy) and mammalian cell data [34,60]. Those studies may have overestimated the number of DSBs channeled to HR repair since higher doses of irradiation were not tested. Moreover, recovery of CO numbers to wild type levels in *com-1* mutants, that lack normal levels of DSB end resection, can only be achieved with higher doses of gamma irradiation (70 Gy) similar to our observations [61]. Our results suggest that high doses of gamma irradiation produce an excess of DNA damage that is either channeled into different repair pathways or repaired by HR in a timely manner. In addition, endogenous meiotic DSBs occur asynchronously, and therefore the analysis of fixed samples only provides a snapshot of a subset of DSB repair events, whereas IR-induced DSBs are produced simultaneously allowing us to measure mostly synchronous processing of DSBs in all regions. High doses of irradiation could also alter chromatin configuration. The enrichment of RAD-51 foci at the center following 100 Gy could be due to alterations in chromatin organization (i.e., an expansion of the central region) similar to what is observed in mutants for chromatin modifiers (Figs 1G and 4C). Radiation stress in human cells increases transcription via the MAPK pathway [62]. A similar scenario could be occurring in the central region of the chromosomes, since in *C*. *elegans* this corresponds to high gene density areas [8,40].

In *rad-54* mutants we observed an unbiased distribution of RAD-51 foci. These results suggest that SPO-11 dependent and independent DSBs in wild type worms can occur in both center and arm locations of the chromosomes, but either the pathway or timing of their repair dramatically biases their processing via RAD-51 towards the arms. One explanation for an unbiased distribution of RAD-51 foci is that when the HR pathway is blocked the lack of CO designation fails to activate a feedback loop to prevent additional DSB formation, thereby resulting in excess DSBs [63]. Alternatively, when there is an excess number of breaks all repair pathways may be used to their maximum capacity.

### Chromatin configuration affects center versus arms boundaries in DSB processing

We observed that altering the chromatin landscape either by affecting chromosome condensation or regulation of histone methylation in the *dpy-28*, *met-2*, and *set-25* mutants can change the distribution of RAD-51 foci along the length of the chromosomes (Fig 6). Our results show that *dpy-28* mutants present a less off-center biased RAD-51 distribution than wild type (Fig 3B). However, this redistribution of breaks does not depend on the extension of the axial length per se since in the *mnT12* IV;X chromosome fusion strain the distribution of RAD-51 foci is similar to wild type (Fig 3F). Therefore, increasing axial length does not contribute to a change in the distribution of breaks (RAD-51 marked sites are still primarily detected on the “new” arms of this fused pair of chromosomes), probably because chromatin loop size is not affected in the strain carrying the chromosome fusion. Another possibility is that in hermaphroditic worms carrying the IV;X fusion, epigenetic marks present on the X chromosome may be extending into the attached IV, as shown in males [64], affecting the boundaries of arms versus center regions. Chromosome length can also influence the number of breaks, with fewer breaks along shorter chromosomes in *C*. *elegans* (Fig 3D, [36]). However, short chromosomes could upregulate DSB levels for CO assurance as shown in budding yeast [65]. Taken together, we propose that changes in chromatin loop density due to the extension of axis length in *dpy-28* mutants, could increase DSB levels at the center region of the chromosomes, allowing for more strand invasion and repair of endogenous DSBs by the HR pathway in that region.

In contrast, *met-2* and *set-25* single and double mutants exhibit a biased distribution of RAD-51 foci towards the center of the bivalents (Fig 4). MET-2 and SET-25 proteins deposit H3K9me1/me2 and H3K9me3, respectively, in heterochromatic regions corresponding to the distal arms of the chromosomes where CO recombination takes place [40–43]. Moreover, heterochromatic regions associated with these histone methylation marks localize near the nuclear envelope and nuclear pores in embryos, suggesting that the 3D localization of the arms of the chromosomes could also contribute to regulation of DSB processing in the germline [43]. One caveat is that the redistribution of RAD-51 foci in these backgrounds may arise in part from the higher levels of RAD-51 foci observed in *met-2* and *set-25* single and double mutants (Fig 4B). However, this increase in RAD-51 foci partly stems from R-loops, derepression of satellite repeat transcription, and replication stress in these mutants [41,66], that could be regulated differently than IR-induced exogenous breaks. Furthermore, *him-17* mutants lack the same chromatin-associated H3K9me2 mark, have reduced levels of RAD-51 foci [19] and still show the same enrichment of RAD-51 foci at the center of the chromosomes (Fig 4A, [27]). This implies that the phenotypes observed for these three chromatin modifiers are dependent on their role in H3K9 methylation. In summary, repressive epigenetic marks enriched at the arms of the chromosomes could serve as a signal for the preferential recruitment of HR proteins that participate in resection and strand-invasion, whereas the loss of these marks could redistribute recruitment of HR proteins along the length of the chromosomes.

### DSB detection by cKU-70/80 but not NHEJ influences RAD-51 loading

During meiosis HR is the predominant pathway for DSB repair, which ensures accurate transmission of the genetic information to the next generation [67]. Whenever HR is unavailable or misregulated, the NHEJ pathway participates in the repair of SPO-11-dependent and exogenous breaks [25,26,68], implying that the NHEJ repair machinery is active and accessible during meiosis. However, it is unclear whether there is competition between HR and NHEJ proteins for the detection and repair of DSBs in wild type conditions. Our results help clarify the roles of HR and NHEJ since absence of cKU-70/80 dramatically alters the distribution of RAD-51 foci along the chromosomes (Fig 5). Furthermore, *cku-70/80* genes are homogeneously expressed during meiosis with a peak in early to mid-pachytene [69], and are involved in blocking DSB resection when COM-1 is absent [61]. Interestingly, DSB repair by NHEJ is not involved in the regulation of RAD-51 foci position, since mutants of *lig-4*, the ligase that joins blunt DSB ends in NHEJ, do not alter RAD-51 localization along chromosomes (Fig 5B and 5D). A different function of the cKUs and LIG-4 has been observed in *C*. *elegans com-1* mutants where the absence of Cos can be suppressed when depleting *cku-70/80* but not *lig-4* [61]. Similarly in yeast, loss of Lig4 has a mild effect on the number of Mre11-dependent resected DSBs, whereas absence of the Ku proteins shows a strong increase of DSB resection in G1 [70]. One possible explanation is that SPO-11-dependent DSBs in *C*. *elegans* are homogeneously distributed, but cKU-70/80 could block their resection more frequently in the center region of the chromosomes. Other pathways could therefore be repairing the DSBs on the center region, such as single-strand annealing (SSA), mediated mainly by RAD-52 and XPF-1, and alternative end joining, such as microhomology-mediated end joining (MMEJ) modulated by POLQ-1 [71]. Even though XPF-1 has been involved in many meiotic aspects [72–74,51,75], SSA and MMEJ seem to play a less important role in DSB repair during meiosis than NHEJ when HR is absent [76,77]. Another possibility is that the cKUs might regulate either the timing of DSB processing or the recruitment of other regulators, and in the absence of the cKUs, RAD-51 foci are preferentially on the center of the chromosomes because incorrect proteins are being recruited to places where normally HR is not used. Lastly, a recent study has shown that DSBs formed in mid to late pachytene are preferentially resected by EXO-1 and DNA-2 in *C*. *elegans* [78], so it is possible that DSBs at the center of the chromosomes might be processed by the EXO-1-DNA-2 machinery that acts late in pachytene to form COs.

In summary, we uncovered different factors that regulate RAD-51 foci distribution along the length of the chromosomes in *C*. *elegans*, and bias RAD-51 strand invasion preferentially at the distal arms of the chromosomes (Fig 6). Chromatin modifications can define regions of high and low DSB levels and recruit different regulators. Chromosome compaction in an array of chromatin loops also contributes to these arm and center region limits. Higher levels of DSB formation can bypass these limits on RAD-51 distribution, and DSB early recognition could be the effector of this regulation. Multiple layers of regulation contribute to an early bias in DSB strand invasion and the late bias in CO distribution. These factors ultimately prevent formation of COs at the center of the chromosomes that otherwise would lead to abnormal symmetric bivalents [1] and the missegregation of chromosomes in meiosis.

## Materials and methods

### Worm strains and growth conditions

The N2 Bristol strain was used as the wild-type background. *C*. *elegans* strains were cultured at 20°C using standard growth conditions [79]. The following mutations and chromosome rearrangements were used: *spo-11(ok79)* [31], *rad-54(ok615)* [36], *dsb-1(tm5034)* [35], *him-17(ok424)* [19], *met-2(n4256)*, *set-25(n5021)* [42], *dpy-28(s939)* [36], *lig-4(ok716)* [44], *cku-80(ok861)* [44] and *mnT-12* (IV:X) [38].

### RNA interference

RNAi was performed using the Ahringer RNAi library [80] sequence-verified clones as in [81] with modifications [82]. L4-stage animals (P0s) were grown at room temperature on plates with HT115 bacteria expressing either the empty vector (EV) or the RNAi construct pL4440 plasmids. L4 progeny (F1s) were used for irradiation and immunofluorescence experiments. 24h post-L4 animals were collected in 100 μl of Trizol (Invitrogen), followed by RNA extraction following the manufacturer’s instructions. cDNA production was performed using iScript (BioRad). RNAi effectiveness was determined by assaying the expression of the transcript being depleted in 4 to 5 individual animals and a mix of worms as shown in S5 Fig. The following primers were used:

cku-70_RT_fw CTCGGAGACCGAGGGACTCG

cku-70_RT_rv CGTTTCTCGTCCTTATCCTGTGGC

lig-4_RT_fw CGGATTACGAGAGGTTCAGAAAGTTCG

lig-4_RT_rv CCCAAATAGCACAAGCAGAAGGCG

rad-54_RT_fw CCACAAGCTCTACTGCTCCAAC

rad-54_RT_rv TACCACGTGCACCTTTCGATC

gpdh-1_RT_fw GCAATTGTTGGCGGTGGAAACTG

gpdh-1_RT_rv CTGAGGGATCCCTTGGTGTGGG

### Gamma irradiation

22-24h post-L4 animals were exposed to 0, 10, 20, 30, 60 or 100 Gy from a Cs^137^ source. Irradiated (+IR) and untreated (-IR) control worms were dissected 1 hr post-irradiation, when maximum levels of RAD-51 foci are detected in wild type [34,83].

### Antibodies and Immunofluorescence

Dissection and preparation of whole-mounted *C*. *elegans* gonads was performed as in [29] with modifications. After 1 hr blocking with 0.5% BSA, slides were incubated overnight at 4°C with primary antibodies: guinea pig α-HTP-3 (1:500, [84]), rabbit α-RAD-51 (1:10,000; Novus Biological (SDI)), mouse α-AcK (1:1000; Cell Signaling Technology), goat α-SYP-1 (1:2000, [63]), and guinea pig α-ZHP-3 (1:500, [85]). Secondary antibody incubation was performed at room temperature for 2h with: α-rabbit Cy3 (1:200), α-guinea pig Cy3 (1:200), α-mouse Cy5 (1:100), α-guinea pig Alexa 488 (1:500), and α-goat Alexa 488 (1:500) from Jackson ImmunoResearch Laboratories (West Grove, PA), AffiniPure IgG (H+L) with minimum cross reactivity.

### Microscopy and imaging

Immunofluorescence images were acquired with an IX-70 microscope (Olympus) and a cooled CCD camera (model CH350, Roper Scientific) controlled by the DeltaVision system (Applied Precision). Optical sections were collected at 0.2μm increments with a 100x objective (N.A. 1.4) and additional amplification from the 10X oculars. SoftWorx 3.3.6 software from Applied Precision was used for image acquisition and deconvolution. Image processing was performed using Fiji ImageJ software [86].

### RAD-51 quantification

Number of RAD-51 foci per nucleus was quantified in 3D images in each zone as in [29] using Fiji. Five to six gonads from at least 2 independent biological replicate experiments were quantified per genotype and/or condition (S1 File).

### Chromosome linearization

Chromosomes were computationally straightened by tracing either the HTP-3 axis signal (RAD-51) or SYP-1 signal (ZHP-3) in 3D nuclei with Priism [87] as in [37]. The total length of each chromosome was measured and divided into 3 equally sized regions (arm, center, and arm). Only chromosomes for which both ends were clearly identifiable were analyzed. The position of RAD-51 or ZHP-3 foci on either the arms or the center of the chromosomes was scored, and the presence or absence of the Pan Acetylation K signal was used to determine whether the chromosome corresponded to an autosome or the X chromosome, respectively. In the *mnT12* background, the IV;X fused chromosomes were distinguished by both the absence of Pan Acetylation signal in part of the bivalent and the length (doubled) of the fused bivalent. Raw data of the total length per chromosome and number of RAD-51 foci per region is shown in S2 File.

### Statistics

GraphPad Prism software was used for statistical comparisons and the generation of graphical representations of the data. A two-sided non-parametric Mann Whitney U-test was used to perform comparisons of RAD-51 foci per nucleus and number of RAD-51 foci per chromosome, between wild type and mutant backgrounds. Fisher’s exact test was used to compare the distribution of RAD-51 foci along chromosomes (arm versus center) in wild type and mutants, and between different zones along the germline. Chromosome lengths between wild type and different mutant backgrounds were compared using an independent sample two-sided t-Student test. Microsoft Excel 365 was used to calculate regression coefficients and to represent the correlations between the number of RAD-51 foci per chromosome and the length of the chromosome.

## Supporting information

S1 Fig Levels and chromosomal distribution of RAD-51 foci in wild type and *dsb-1* mutants (+/–IR). **(A)** High-resolution images representative of mid-pachytene nuclei (zone 5) immunostained for RAD-51 (magenta) and co-stained with DAPI (blue). To score the number of RAD-51 foci in cases when the foci overlapped and formed tracks, we used the diameter of a single focus (based on the mean length identified from multiple experiments). Scale bar, 2 μm. **(B)** Top, high-magnification image of a full-projection of a mid-pachytene nucleus co-stained with anti-HTP-3 (green), to trace chromosome axes, anti-RAD-51 (magenta), to mark DSB repair sites, and AcK (blue) to distinguish the X-chromosome. The X-chromosome (no signal for AcK) selected for linearization is shown with a yellow dashed line. Bottom, chromosome computationally linearized using PRIISM Software. Linearized chromosomes were divided into three equal length portions referred to as arms and center. Scale bar, 2 μm. **(C)** High-resolution images of mid-pachytene nuclei (zone 5) from wild-type animals exposed to different doses of IR immunostained for RAD-51 (magenta) and co-stained with DAPI (blue). Scale bar, 2 μm. **(D)** Histogram shows the distribution of RAD-51 foci at the center versus the arms of the chromosomes in the early pachytene (EP, left) and late pachytene (LP, right) stage in wild-type animals exposed to the indicated doses of IR (x-axis). Distribution of RAD-51 foci along the chromosomes is biased (majority of the RAD-51 foci are present at the arms of the chromosomes) from 0 to 30 Gy; however, this distribution changes significantly to a more even distribution at 60 Gy and is enriched at the center of the chromosomes at 100 Gy. Percentages are indicated above each bar graph. *p<0.05, **p<0.01, ***p<0.001, ****p<0.0001 by Fisher’s exact test. **(E)** Histogram depicts the mean number of RAD-51 foci/nucleus observed in different zones of wild-type and *dsb-1* mutant germlines +/–IR (60Gy). X-axis shows the position along the germline. PMT-premeiotic tip nuclei in mitosis, L/Z-meiotic nuclei in leptotene/zygotene, EP- nuclei in early pachytene, MP- nuclei in mid-pachytene, and LP- nuclei in late pachytene. At least 6 gonads were scored per genotype. Mean number is indicated above each bar graph. Error bars represent SEM from technical repeats for each of two biological replicates. **p<0.01. Comparisons that scored significant between wild-type + 60 Gy and *dsb-1* + 60 Gy are indicated. **(F)** Histogram shows a similar distribution of RAD-51 foci at the center versus the arms of the chromosomes throughout pachytene between wild-type and *dsb-1* mutants exposed to IR (60 Gy). Comparisons among different zones scored not significant by Fisher’s exact test, see S2 File.
(TIF)

S2 Fig RAD-51 foci levels and distribution in wild type, *rad-54*, *dpy-28*, and *mnT12 (*+/–IR). **(A)** Histogram shows the distribution of RAD-51 foci at the center versus the arms of the chromosomes in *rad-54* mutants compared to wild-type exposed to IR (60 Gy). *rad-54* mutants show similar distribution of RAD-51 foci compared to wild-type throughout pachytene. Comparisons among different zones scored not significant by Fisher’s exact test, see S2 File. **(B)** Histogram depicts the mean number of RAD-51 foci/nucleus observed in different zones of *dpy-28* mutant germlines compared to wild-type exposed to IR (60Gy). X-axis shows the position along the germline: PMT-premeiotic tip (germ cells in mitosis), L/Z- leptotene/zygotene, EP- early pachytene, MP- mid-pachytene, and LP- late pachytene. Mean number is indicated above each bar. At least 6 gonads were scored per genotype. Error bars represent SEM from technical repeats for each of two biological replicates. ****p<0.0001 by the two-tailed Mann-Whitney test, 95% C.I. **(C)** Histogram shows the distribution of RAD-51 foci at the center versus the arms of the chromosomes in *dpy-28* mutants compared to wild-type +/- IR (60 Gy). A similar distribution of RAD-51 foci is observed in *dpy-28* mutants compared to wild-type throughout pachytene. Percentages are indicated above each bar graph. *p<0.05 by Fisher's exact test. **(D)** Histogram depicts the mean number of RAD-51 foci/nucleus observed in different zones of *mnT12* mutant germlines exposed to IR (60 Gy) compared to wild-type worms. X-axis shows the position along the germline. Mean number is indicated above each bar graph. At least 6 gonads were scored per genotype. Error bars represent SEM from technical repeats for each of two biological replicates. Comparisons between wild-type and *mnT12* mutant worms scored not significant by the two-tailed Mann-Whitney test, 95% C.I., see S1 File. **(E)** Histogram shows the distribution of RAD-51 foci in the center versus the arm regions of the chromosomes in *mnT12* mutants exposed to IR (60 Gy) compared to wild-type worms. *mnT12* mutant worms show similar distribution of RAD-51 foci compared to wild-type throughout pachytene. Comparisons among different zones scored not significant by Fisher’s exact test, see S2 File.
(TIF)

S3 Fig RAD-51 foci levels and distribution in *him-17*, *met-2*, *set-25*, and *met-2 set-25* compared to wild type (+/–IR). **(A)** Table summarizes the distribution of RAD-51 foci at the arms versus the center of the chromosomes in the indicated genotypes +/- IR making the distinction between autosomes and X chromosomes. **(B)** Histogram depicts the mean number of RAD-51 foci/nucleus observed in different zones of *him-17* mutant germlines +/- IR (60Gy) compared to wild-type. X-axis shows the position along the germline. Mean number of RAD-51 foci is indicated above each bar graph. At least 6 gonads were scored per genotype. Error bars represent SEM from technical repeats for each of two biological replicates. **p<0.01, ***p<0.001 ****p<0.0001. Comparisons that scored significant by the two-tailed Mann-Whitney test, 95% C.I. between wild-type + 60 Gy and *him-17* + 60 Gy are indicated**. (C)** Table summarizes the distribution of RAD-51 foci at the arms versus the center regions of the chromosomes for the indicated genotypes exposed to IR (60 Gy). EP, Early pachytene; MP, Mid-pachytene; LP, Late pachytene. **(D)** Histogram depicts the mean number of RAD-51 foci/nucleus observed in different zones of *met-2*, *set-25*, and *met-2 set-25* mutant germlines exposed to IR (60 Gy) compared to wild-type. X-axis shows the position along the germline. Mean number of RAD-51 foci is indicated above each bar graph. At least 6 gonads were scored per genotype. Error bars represent SEM from technical repeats for each of two biological replicates. ****p<0.0001. Comparisons that scored significant by the two-tailed Mann-Whitney test, 95% C.I. between wild-type + 60 Gy and *met-2*, *set-25*, and *met-2 set-25* + 60 Gy are indicated**. (E)** Table summarizes the distribution of RAD-51 foci at the arms versus the center regions of the chromosomes for the indicated genotypes, making the distinction between autosomes and X chromosomes.
(TIF)

S4 Fig RAD-51 foci levels and distribution in wild type, *cku-80*, and *lig-4* (+/- IR). **(A)** Histogram depicts the mean number of RAD-51 foci/nucleus observed in different zones of *cku-80* and *lig-4* mutant germlines + IR (60Gy) compared to wild-type. X-axis shows the position along the germline. Mean number is indicated above each bar graph. At least 6 gonads were scored per genotype. Error bars represent SEM from technical repeats for each of two biological replicates. ***p<0.001, ****p<0.0001 by the two-tailed Mann Whitney test, 95% C.I. **(B)** Table summarizes the distribution of RAD-51 foci at the arms versus center of the chromosomes for the indicated genotypes +/- IR making the distinction between autosomes and X chromosomes. EP, Early pachytene; MP, Mid-pachytene; LP, Late pachytene. **(C)** Table summarizes the distribution of RAD-51 foci at the center versus the arms of the chromosomes for the indicated genotypes +IR (60 Gy). **(D)** Histogram shows the distribution of ZHP-3 foci at the arms versus center of the chromosomes in *cku-80* mutants compared to wild-type. Percentages are indicated above each bar graph. ***p<0.001 by Fisher’s exact test.
(TIF)

S5 Fig Assessing RNAi depletion. RT-PCR using primers specific for *cku-70*, *lig-4*, *rad-54*, and *gpdh-1* as a control to show the level of RNAi depletion achieved by feeding for *cku-70*, *lig-4*, and *rad-54* compared to worms fed with empty vector (EV). Each lane corresponds to a sample coming from a single worm except for the last lane which in each case corresponds to a pool of 10 worms.
(TIF)

S1 File Raw data of the total number of RAD-51 foci counts. Number of RAD-51 foci scored per nucleus in each zone is shown in the indicated genotypes.
(XLSX)

S2 File Raw data of the total length per chromosome and number of RAD-51 foci per region. (XLSX)
