# Supplementary figures and images for: Chromatin landscape, DSB levels, and cKU-70/80 contribute to patterning of meiotic DSB processing along chromosomes in C. elegans
Source: PLoS Genet. 2023 Jan 27;19(1):e1010627. doi: 10.1371/journal.pgen.1010627 (PMC9907818; doi:10.1371/journal.pgen.1010627)

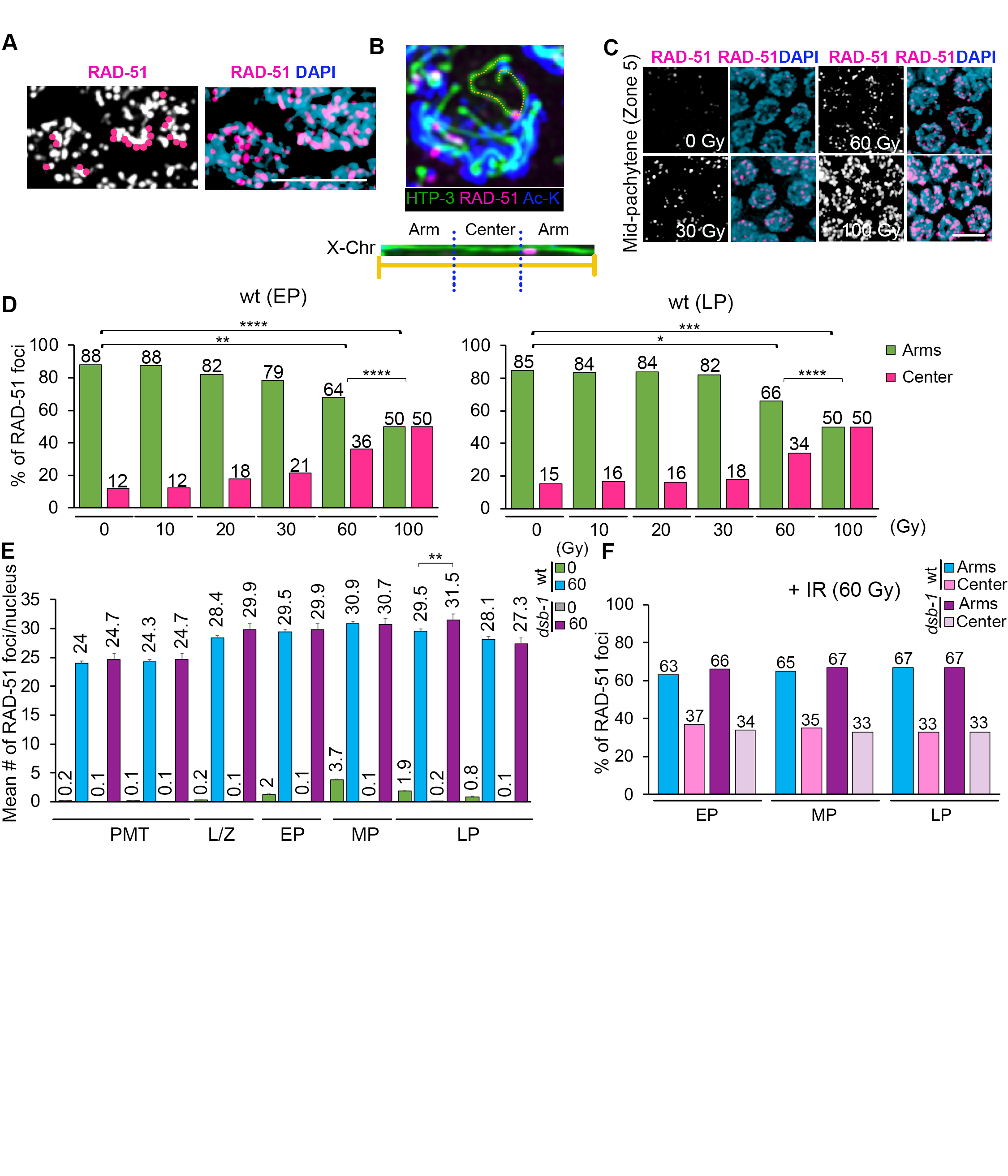

Supplement: S1 Fig — (A) High-resolution images representative of mid-pachytene nuclei (zone 5) immunostained for RAD-51 (magenta) and co-stained with DAPI (blue). To score the number of RAD-51 foci in cases when the foci overlapped and formed tracks, we used the diameter of a single focus (based on the mean length identified from multiple experiments). Scale bar, 2 μm. (B) Top, high-magnification image of a full-projection of a mid-pachytene nucleus co-stained with anti-HTP-3 (green), to trace chromosome axes, anti-RAD-51 (magenta), to mark DSB repair sites, and AcK (blue) to distinguish the X-chromosome. The X-chromosome (no signal for AcK) selected for linearization is shown with a yellow dashed line. Bottom, chromosome computationally linearized using PRIISM Software. Linearized chromosomes were divided into three equal length portions referred to as arms and center. Scale bar, 2 μm. (C) High-resolution images of mid-pachytene nuclei (zone 5) from wild-type animals exposed to different doses of IR immunostained for RAD-51 (magenta) and co-stained with DAPI (blue). Scale bar, 2 μm. (D) Histogram shows the distribution of RAD-51 foci at the center versus the arms of the chromosomes in the early pachytene (EP, left) and late pachytene (LP, right) stage in wild-type animals exposed to the indicated doses of IR (x-axis). Distribution of RAD-51 foci along the chromosomes is biased (majority of the RAD-51 foci are present at the arms of the chromosomes) from 0 to 30 Gy; however, this distribution changes significantly to a more even distribution at 60 Gy and is enriched at the center of the chromosomes at 100 Gy. Percentages are indicated above each bar graph. *p<0.05, **p<0.01, ***p<0.001, ****p<0.0001 by Fisher’s exact test. (E) Histogram depicts the mean number of RAD-51 foci/nucleus observed in different zones of wild-type and dsb-1 mutant germlines +/–IR (60Gy). X-axis shows the position along the germline. PMT-premeiotic tip nuclei in mitosis, L/Z-meiotic nuclei in leptotene/zygotene [file pgen.1010627.s001.tif]

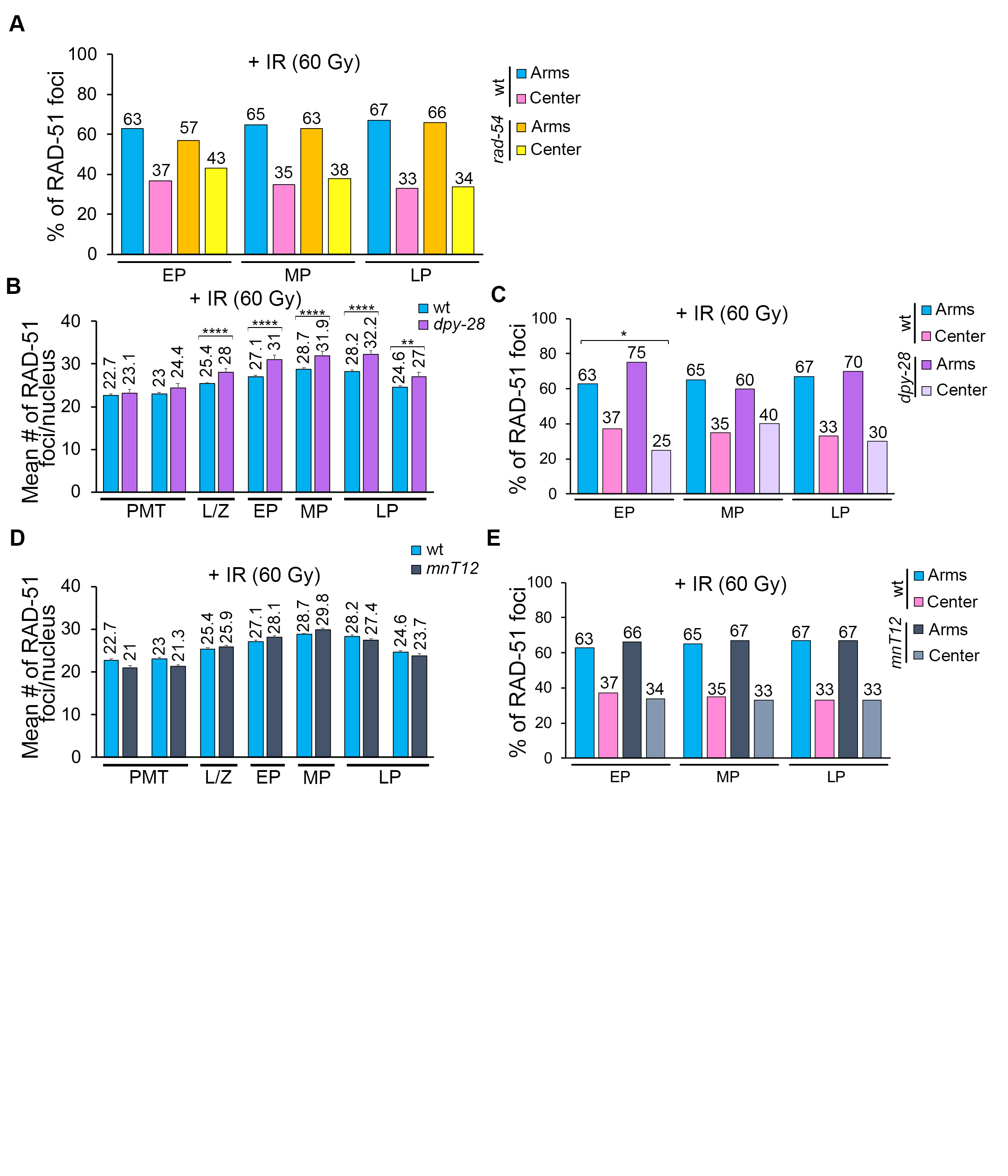

Supplement: S2 Fig — (A) Histogram shows the distribution of RAD-51 foci at the center versus the arms of the chromosomes in rad-54 mutants compared to wild-type exposed to IR (60 Gy). rad-54 mutants show similar distribution of RAD-51 foci compared to wild-type throughout pachytene. Comparisons among different zones scored not significant by Fisher’s exact test, see S2 File. (B) Histogram depicts the mean number of RAD-51 foci/nucleus observed in different zones of dpy-28 mutant germlines compared to wild-type exposed to IR (60Gy). X-axis shows the position along the germline: PMT-premeiotic tip (germ cells in mitosis), L/Z- leptotene/zygotene, EP- early pachytene, MP- mid-pachytene, and LP- late pachytene. Mean number is indicated above each bar. At least 6 gonads were scored per genotype. Error bars represent SEM from technical repeats for each of two biological replicates. ****p<0.0001 by the two-tailed Mann-Whitney test, 95% C.I. (C) Histogram shows the distribution of RAD-51 foci at the center versus the arms of the chromosomes in dpy-28 mutants compared to wild-type +/- IR (60 Gy). A similar distribution of RAD-51 foci is observed in dpy-28 mutants compared to wild-type throughout pachytene. Percentages are indicated above each bar graph. *p<0.05 by Fisher's exact test. (D) Histogram depicts the mean number of RAD-51 foci/nucleus observed in different zones of mnT12 mutant germlines exposed to IR (60 Gy) compared to wild-type worms. X-axis shows the position along the germline. Mean number is indicated above each bar graph. At least 6 gonads were scored per genotype. Error bars represent SEM from technical repeats for each of two biological replicates. Comparisons between wild-type and mnT12 mutant worms scored not significant by the two-tailed Mann-Whitney test, 95% C.I., see S1 File. (E) Histogram shows the distribution of RAD-51 foci in the center versus the arm regions of the chromosomes in mnT12 mutants exposed to IR (60 Gy) compared to wild-type worms. mnT12 mutant worms sh [file pgen.1010627.s002.tif]

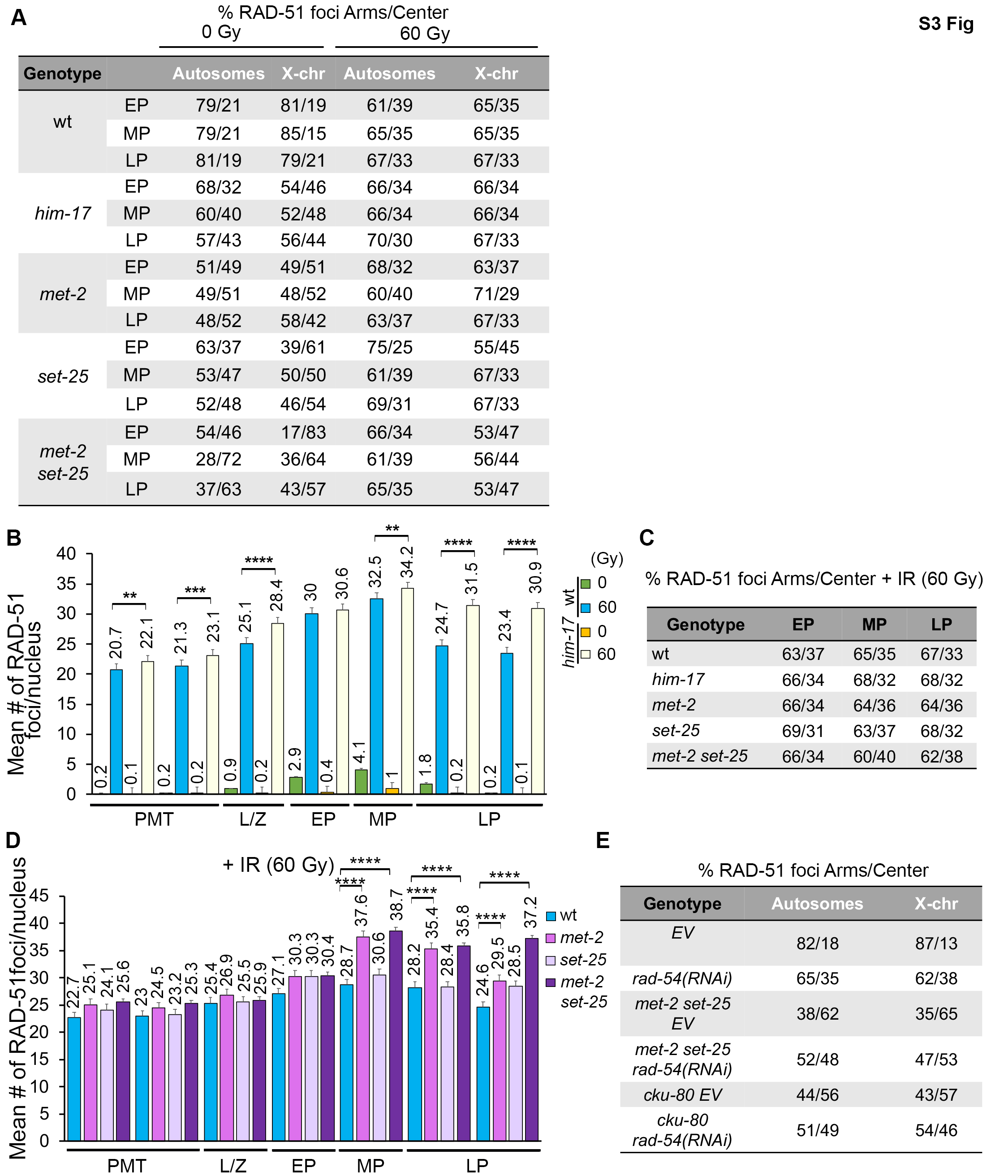

Supplement: S3 Fig — (A) Table summarizes the distribution of RAD-51 foci at the arms versus the center of the chromosomes in the indicated genotypes +/- IR making the distinction between autosomes and X chromosomes. (B) Histogram depicts the mean number of RAD-51 foci/nucleus observed in different zones of him-17 mutant germlines +/- IR (60Gy) compared to wild-type. X-axis shows the position along the germline. Mean number of RAD-51 foci is indicated above each bar graph. At least 6 gonads were scored per genotype. Error bars represent SEM from technical repeats for each of two biological replicates. **p<0.01, ***p<0.001 ****p<0.0001. Comparisons that scored significant by the two-tailed Mann-Whitney test, 95% C.I. between wild-type + 60 Gy and him-17 + 60 Gy are indicated. (C) Table summarizes the distribution of RAD-51 foci at the arms versus the center regions of the chromosomes for the indicated genotypes exposed to IR (60 Gy). EP, Early pachytene; MP, Mid-pachytene; LP, Late pachytene. (D) Histogram depicts the mean number of RAD-51 foci/nucleus observed in different zones of met-2, set-25, and met-2 set-25 mutant germlines exposed to IR (60 Gy) compared to wild-type. X-axis shows the position along the germline. Mean number of RAD-51 foci is indicated above each bar graph. At least 6 gonads were scored per genotype. Error bars represent SEM from technical repeats for each of two biological replicates. ****p<0.0001. Comparisons that scored significant by the two-tailed Mann-Whitney test, 95% C.I. between wild-type + 60 Gy and met-2, set-25, and met-2 set-25 + 60 Gy are indicated. (E) Table summarizes the distribution of RAD-51 foci at the arms versus the center regions of the chromosomes for the indicated genotypes, making the distinction between autosomes and X chromosomes. (TIF) [file pgen.1010627.s003.tif]

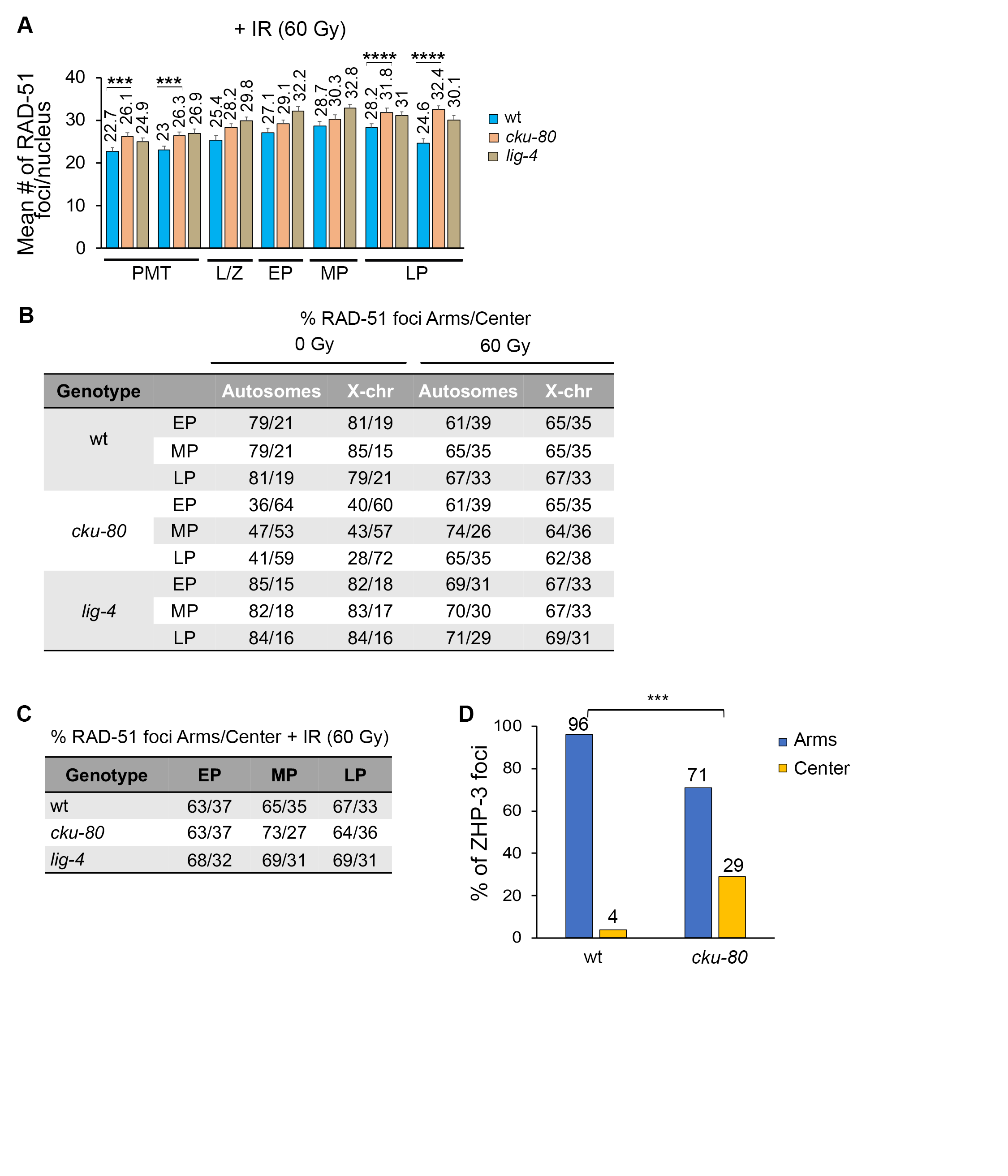

Supplement: S4 Fig — (A) Histogram depicts the mean number of RAD-51 foci/nucleus observed in different zones of cku-80 and lig-4 mutant germlines + IR (60Gy) compared to wild-type. X-axis shows the position along the germline. Mean number is indicated above each bar graph. At least 6 gonads were scored per genotype. Error bars represent SEM from technical repeats for each of two biological replicates. ***p<0.001, ****p<0.0001 by the two-tailed Mann Whitney test, 95% C.I. (B) Table summarizes the distribution of RAD-51 foci at the arms versus center of the chromosomes for the indicated genotypes +/- IR making the distinction between autosomes and X chromosomes. EP, Early pachytene; MP, Mid-pachytene; LP, Late pachytene. (C) Table summarizes the distribution of RAD-51 foci at the center versus the arms of the chromosomes for the indicated genotypes +IR (60 Gy). (D) Histogram shows the distribution of ZHP-3 foci at the arms versus center of the chromosomes in cku-80 mutants compared to wild-type. Percentages are indicated above each bar graph. ***p<0.001 by Fisher’s exact test. (TIF) [file pgen.1010627.s004.tif]

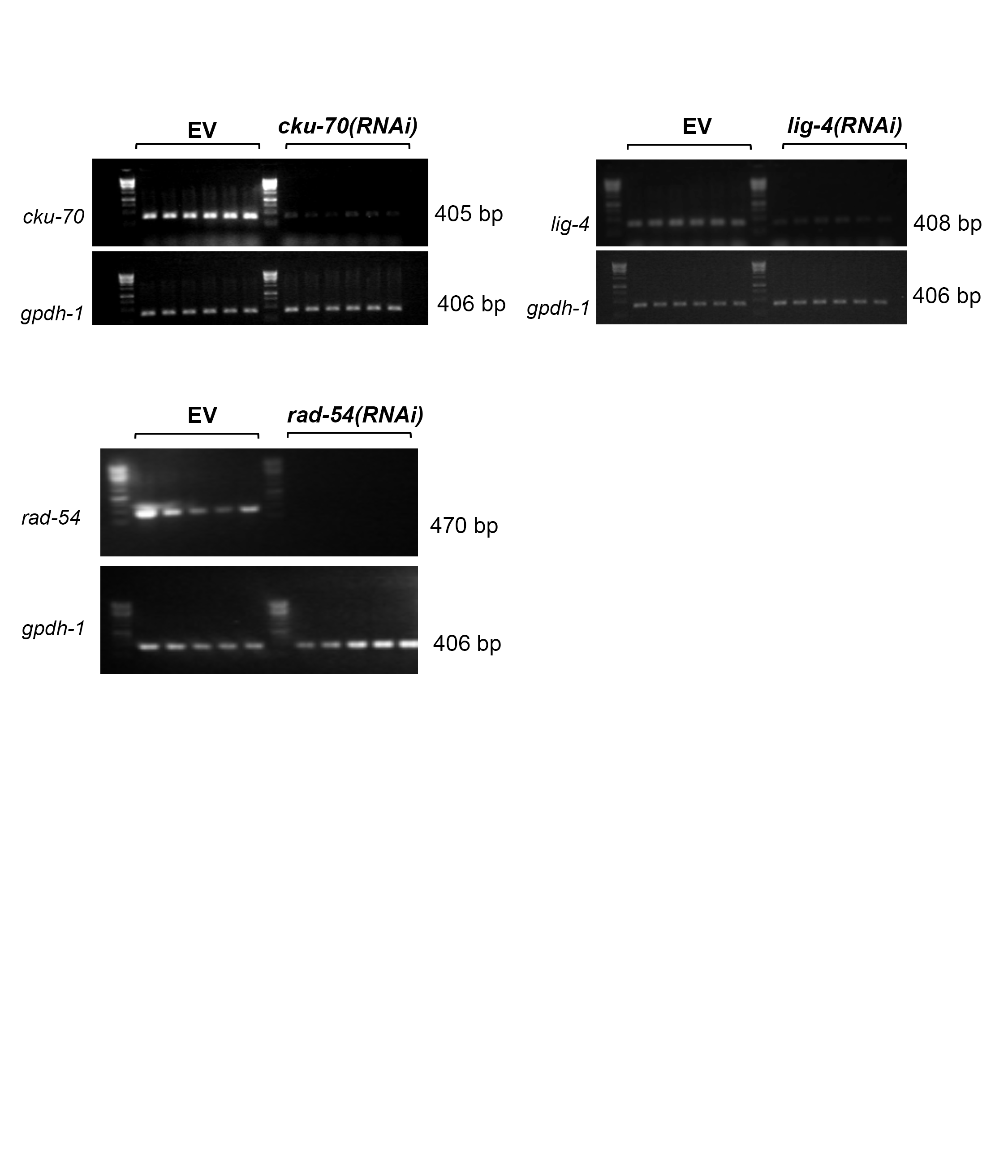

Supplement: S5 Fig — RT-PCR using primers specific for cku-70, lig-4, rad-54, and gpdh-1 as a control to show the level of RNAi depletion achieved by feeding for cku-70, lig-4, and rad-54 compared to worms fed with empty vector (EV). Each lane corresponds to a sample coming from a single worm except for the last lane which in each case corresponds to a pool of 10 worms. (TIF) [file pgen.1010627.s005.tif]
